# Surface‐Capped Protein Nanoparticles for Nonviral Gene Delivery

**DOI:** 10.1002/adma.202521796

**Published:** 2026-03-12

**Authors:** Fjorela Xhyliu, Yao Yao, Yeongun Ko, Grant Grasman, Jeffery E. Raymond, Albert Chang, Yuxuan Deng, Grant Dominic, Michael Triebwasser, Joerg Lahann

**Affiliations:** ^1^ Biointerfaces Institute University of Michigan Ann Arbor Michigan USA; ^2^ Department of Chemical Engineering University of Michigan Ann Arbor Michigan USA; ^3^ School of Dentistry University of Michigan Ann Arbor Michigan USA; ^4^ School of Polymer Science and Engineering Chonnam National University Gwangju South Korea; ^5^ Department of Materials Science and Engineering University of Michigan Ann Arbor Michigan USA; ^6^ Division of Pediatric Hematology and Oncology Department of Pediatrics University of Michigan Ann Arbor Michigan USA

**Keywords:** cell uptake, electrohydrodynamic jet, gene delivery, nanoparticles, pDNA, transfection

## Abstract

Developing simple, safe, and efficient nonviral delivery systems remains a significant challenge in bioengineering. Nanoparticles offer promising gene delivery capabilities with reduced toxicity; however, long‐standing challenges related to effective plasmid encapsulation and delivery exist. Here, we report a novel protein‐based nanoparticle platform prepared by electrohydrodynamic jetting that features effective plasmid encapsulation and release. The protein, serum albumin, acts as the structural component of the nanoparticle and encapsulates the plasmid. To avoid chemical cross‐linking, the protein‐based nanoparticles are surface‐capped via interfacial complexation with a polycationic polymer. Surface‐capped protein nanoparticles (scPNPs) show excellent stability for 12 days at physiological pH values and have exceptionally high payload ratios of 10% to 40% wt/wt, corresponding to 28–99 plasmids per scPNP. The uptake efficiency of scPNPs exceeds 95% and engages macropinocytosis and clathrin‐mediated endocytosis pathways. When optimizing nonviral gene therapies, either the payload or the nanoparticle dosage can be varied. For scPNPs, increasing the nanoparticle dosage is more effective than increasing the payload, resulting in a 1.6‐fold increase in transfection rate for identical plasmid amounts. To demonstrate their translational potential, scPNPs were used to effectively encapsulate messenger RNA (mRNA) and transfect primary human T cells, while maintaining high cell viability. This work both advances the fundamental understanding of design choices when optimizing nanoparticle‐based gene delivery and demonstrates the potential of scPNPs for cell therapies.

## 1 Introduction

Introduction of exogenous DNA into the nucleus and genome editing through the delivery of nucleic acids are of high clinical interest [1]. These types of gene therapies hold great promise in solving critical hereditary diseases and cancers [2]. Successful delivery of nucleic acids such as plasmid DNA (pDNA) or messenger RNA (mRNA) into cells requires protection from nuclease degradation, enhanced half‐life, and effective internalization by target cells [3]. Currently, engineered viral vectors (e.g., adenoviruses, retroviruses) are the most common carriers for gene delivery [4]. However, viral vectors suffer from limited cargo capacity, high scale‐up costs, and the risk of severe immune responses [5, 6, 7]. Thus, there is a critical need to find alternatives to viral vectors. A wide range of nonviral gene delivery strategies has been devised based on synthetic materials such as polymers (e.g., polyethylenimine (PEI), poly(β‐amino esters)) or lipids (e.g., lipofectamine, ionizable lipids) [3]. Nonviral gene therapies using nanoparticles have been extensively investigated to expand applications into a broad range of clinical environments [3, 8]. However, there are still critical challenges when it comes to the successful delivery of nucleic acids into target cells. These include nanoparticle stability, biosafety, successful internalization of the nucleic acids, and gene transfer efficiency (e.g., endosomal escape and nuclear trafficking) [3, 9]. The first bottleneck in developing nonviral vectors is the packaging of long anionic and hydrophilic nucleic acids into a small particle, i.e., DNA complexation and condensation [10]. The second crucial step is the effective delivery of the therapeutic nucleic acid into target cells [11].

A critical use case for high‐capacity nonviral gene delivery vectors is the genetic engineering of primary T cells for expression of a chimeric antigen receptor (CAR) targeted to a cancer antigen. Conventional approaches, such as lipid nanoparticles or electroporation, are often plagued by low transfection efficiencies and/or high toxicity [12, 13]. It is well established that primary T cells inefficiently internalize nanoparticles and possess high nuclease activity, presenting substantial challenges for nonviral delivery strategies [14, 15]. Robust gene transfer to primary human T cells is thus often considered the gold standard for next‐generation nonviral gene therapies [16].

PEI has long served as a convenient laboratory benchmark for nonviral gene delivery because of its robust transfection efficiency and commercial availability. Although newer platforms such as lipid nanoparticles, poly(β‐amino esters), and cell‐penetrating peptide conjugates have shown superior in vivo efficacy and reduced toxicity, PEI remains one of the most widely used polymers for nonviral gene therapy [17, 18, 19]. PEI condenses the nucleic acid (e.g., mRNA, pDNA) via electrostatic interactions between negatively charged phosphate groups and positively charged amine groups (primary, secondary, and tertiary). The major shortfall of using PEI in gene delivery is that high transfection efficiency is often associated with unfavorable cytotoxicity profiles [20].

Alternatively, protein nanoparticles have been advanced due to their biocompatibility and biodegradability, which are important factors when considering the design of therapeutic carriers [21]. Abraxane, an albumin‐bound paclitaxel, is an early FDA‐approved example of a protein nanoparticle formulation [22]. However, beyond the delivery of hydrophobic drugs [23], protein nanoparticles have not yet been widely employed for the delivery of biomacromolecules, including therapeutic proteins, mRNA, and plasmids.

Electrohydrodynamic (EHD) jetting is a process that utilizes high voltages to atomize dilute solutions of macromolecules, which then coalesce and produce nanometer‐sized particles with narrow size distributions (polydispersity index, PDI < 0.1) [24]. Our group has shown that EHD jetting can be utilized to fabricate protein‐based nanoparticles from a variety of proteins, including albumin, hemoglobin, transferrin, mucin, insulin, catalase, and even protein mixtures [25]. Previous studies from our group have demonstrated the feasibility of using chemical cross‐linking via glutaraldehyde or an N‐hydroxylsuccinimide (NHS) bifunctional polyethylene glycol (PEG) (NHS–PEG–NHS) to stabilize protein nanoparticles [26, 27]. Specifically, our lab fabricated protein‐based nanoparticles for the delivery of siRNA to glioblastoma [24, 28], in which the siRNA was complexed with PEI prior to EHD jetting. However, the same formulation would not be optimal for plasmid delivery because each polynucleic acid requires different carrier properties [29]. DNA has many binding sites, allowing it to bind strongly to polyamines via electrostatic forces, and it will only dissociate in the presence of competitor ions [30]. Compared to siRNA, pDNA binds more strongly to PEI and forms polyplexes with increased stability [31, 32, 33]. However, studies have shown that excessively strong polyplexes may interfere with the intracellular release of the plasmid, reducing their efficiency [34, 35]. In addition, the covalent cross‐linking of the proteins in the nanoparticles results in mesh sizes of a few nanometers [26], which will prevent the plasmid from being released from the nanoparticle matrix.

In contrast, the nanoparticle platform developed in this study, i.e., surface‐capped protein nanoparticles (scPNPs), does not require chemical cross‐linkers and plasmid condensation via PEI. In contrast, the role of the PEI here is to stabilize the as‐jetted protein nanoparticles through interfacial complexation [36, 37, 38] to avoid their rapid degradation under physiological conditions. During nanoparticle collection with a dilute PEI solution, positively charged PEI electrostatically binds to the surface of negatively charged protein nanoparticles, avoiding direct complexation with pDNA encapsulated in the nanoparticle bulk. scPNPs have a weak positive surface charge and are stable in physiological environments. However, at a pH lower than the isoelectric point of albumin, scPNPs rapidly disassemble and release their payload.

## 2 Results and Discussion

### 2.1 Fabrication and Characterization of scPNPs

**FIGURE 1:**
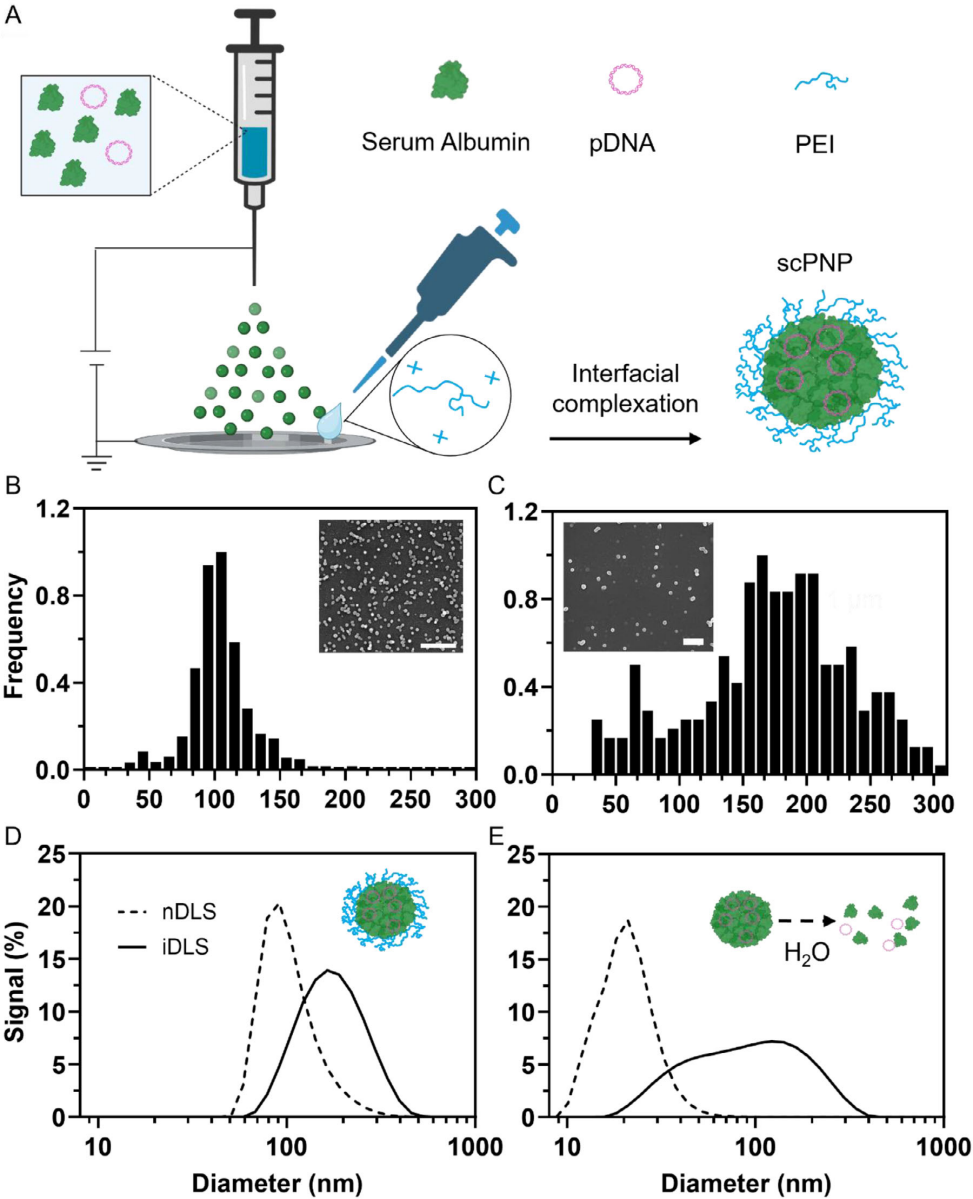
scPNP Synthesis and Characterization. (A) scPNP fabrication scheme. (B) SEM micrograph and diameter characterization of the as‐jetted protein nanoparticles (before surface‐capping). (C) SEM micrograph and diameter analysis after surface capping with PEI and drop‐casting on a silicon chip surface. Frequency data represent individual particles from 5 different images. (D) DLS hydrodynamic diameter distributions of scPNPs (corresponding with 1C), number DLS (nDLS, dashed line), and intensity DLS (iDLS, solid line). (E) DLS hydrodynamic diameter distributions of protein nanoparticles collected with water (disassembled), emphasizing the importance of the interfacial complexation of the polycation to stabilize the protein nanoparticle. SEM scale bars 1 µm. Figure 1A was constructed in and modified from Biorender.com.

The scPNPs were prepared using EHD jetting [24, 25, 26] followed by an interfacial complexation step, also called surface‐capping (Figure 1A). During EHD jetting, rapid solvent evaporation generally yields solid protein/DNA nanoparticles. Unlike previously published protein nanoparticles produced via EHD jetting [24, 25, 26, 39], scPNPs did not contain any reactive cross‐linkers. Instead, the as‐jetted protein nanoparticles were collected in a dilute PEI solution. The scPNPs are stable at physiological pH values for 12 days and only carry a moderate positive charge as indicated by a zeta‐potential of +22.9 ± 2.1 mV.

Interfacial complexation with PEI provides several benefits: (i) it avoids the direct association between PEI with DNA and replaces those interactions with hydrogen bonding between the protein and the DNA during the rapid solvent evaporation step, and (ii) it stabilizes the nanoparticles without any internal cross‐linking.

Albumin has an isoelectric point of 4.7, which implies a net negative charge at physiological pH [40]. Additionally, given the amphiphilic nature of albumin, its hydrophobic and hydrogen‐bonding domains can participate in noncovalent, nonelectrostatic interactions with DNA [41]. Rapid solvent evaporation during EHD jetting creates extremely high local concentrations and shear forces. This induces molecular crowding that can overcome long‐range repulsive electrostatic forces [42, 43], and enables pDNA condensation with albumin. Immediately after jetting, protein nanoparticles were collected using an aqueous solution (Figure 1A). The collection solution contained branched PEI (bPEI) with a molecular weight of 25 kDa, which was included because of its high charge density to allow for rapid association with the surfaces of negatively charged particles [10, 44].

Prior to bPEI collection, nanoparticles exhibited an average dry‐state diameter of 99 ± 5 nm (circularity ∼0.84 ± 0.03) from populations observed via scanning electron microscopy (SEM, Figure 1B). Once stabilized with bPEI, the scPNPs were drop‐cast onto SEM wafers, dried, and assessed again for size and morphology. The scPNPs exhibited slightly larger average dry‐state diameters of 173 ± 14 nm and an unaltered circularity of 0.83 ± 0.02 (Figure 1C). This increase in average diameter is attributed to the addition of a PEI shell and scPNP swelling in the aqueous solution. The scPNP size distribution curves, as assessed by dynamic light scattering (DLS) in the hydrodynamic state, had an average diameter of 177 ± 4 nm with a PDI of 0.13 (number‐ and intensity‐based, Figure 1D). scPNPs maintained their shape after PEI collection and showed exceptional consistency between their SEM diameter and DLS diameters. The dilute solution of bPEI, in addition to the short collection time, allows for surface complexation of individual particles and minimizes particle aggregations, as evidenced by the narrow size distribution. After collection, scPNPs exhibited moderately positive zeta‐potentials of +22.9 ± 2.1 mV, presumably due to the outer PEI layer. Importantly, the polymer capping step was critical in stabilizing the nanoparticles, as uncapped, as‐jetted protein nanoparticles disassembled on contact with water (DLS, Figure 1E).

Using a cyanine‐3 (Cy3)‐labeled pDNA, we determined the encapsulation efficiency to be ∼72% of the total plasmid (Figure S1). Highly efficient plasmid encapsulation and high production yields are important aspects when considering the translation of the technology to the larger production scales required for both preclinical and clinical applications.

### 2.2 Structure, Stability, and Functionality of scPNPs

**FIGURE 2:**
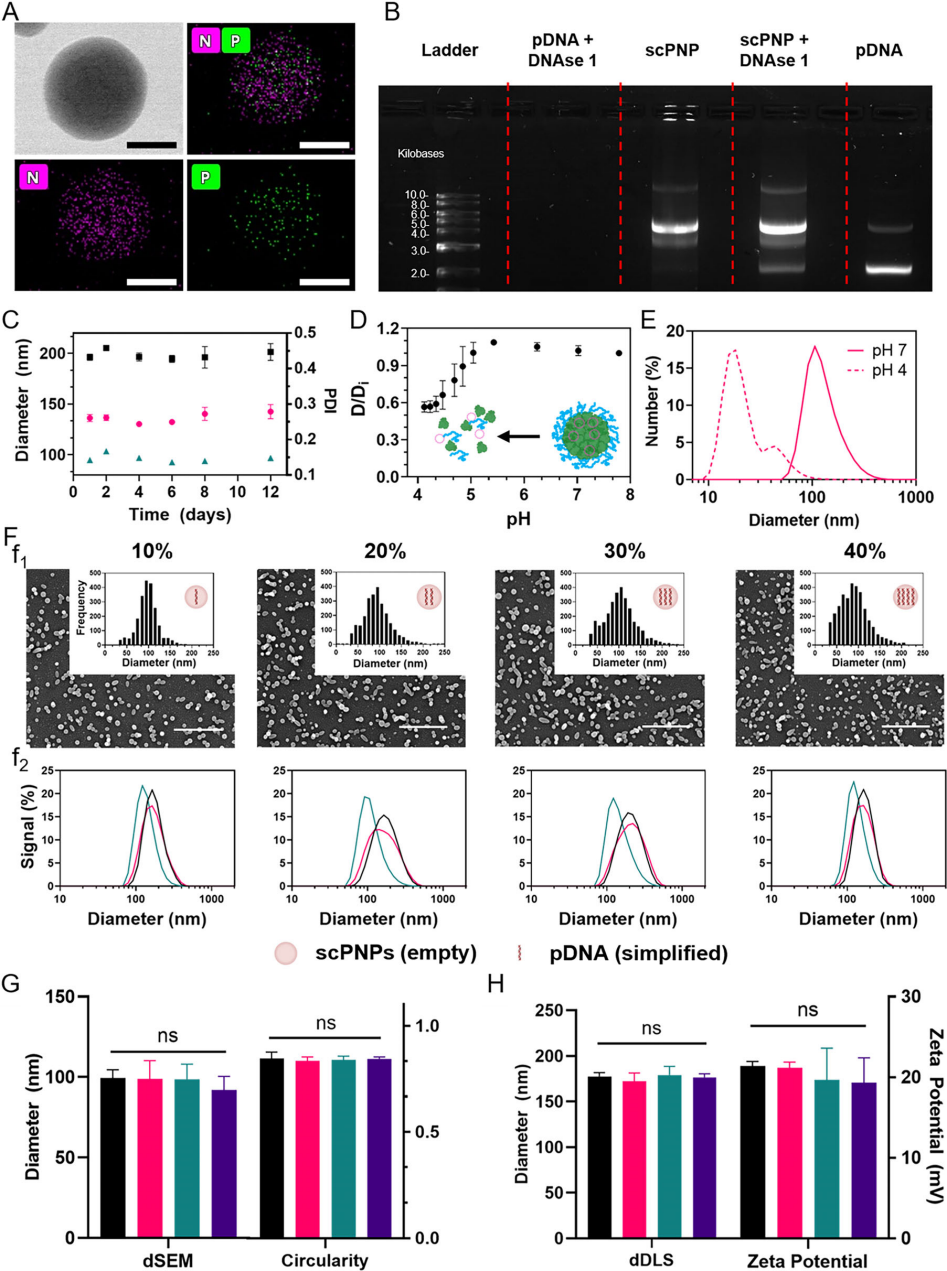
scPNP Homogeneity, Stability, pH Response, and Increased Payload Assessment. (A) STEM‐EDS of the spatial distribution of pDNA (10% w_pDNA_/w_albumin_) in the as‐jetted protein nanoparticles (P = phosphorus, N = nitrogen). Scale bar 50 nm. (B) Gel electrophoresis assay of scPNPs and pDNA with and without incubation with DNAse1. (C) Stability of scPNPs in H_2_O measured via DLS; nDLS (pink), iDLS (black), and PDI (green). The ± in the data represents standard deviation (SD) of *n* = 3 samples. (D) Diameter change of the scPNPs at different pH. The data represent means ± SD of *n* = 3 samples. (E) nDLS curves of scPNPs at pH 7 and pH 4. (F) SEM (f1) and DLS (f2) diameter distributions of scPNPs with varied pDNA loading (w_pDNA_/w_albumin_). SEM scale bar is 1 µm. nDLS (green), iDLS (black), and volume DLS (pink). (G) Dry‐state diameters and circularities of the as‐jetted PNPs with varied pDNA loadings. 10% (black), 20% (pink), 30% (green), and 40% (purple) w/w. (H) Hydrodynamic diameters and zeta potentials of scPNPs with varied pDNA loading. In (D,E) and (G,H), *n* = 3, SD presented as error bars. One‐way analysis of variance test was performed (ns = no significance).

Transmission electron microscopy (TEM) energy dispersive spectroscopy (EDS) was used to confirm nanoparticle structure and morphology. EDS mapping of 10% w/w pDNA‐protein nanoparticles indicated a homogeneous spatial distribution of both the pDNA (phosphorus, P) and protein (nitrogen, N), after jetting (Figure 2A; Figure S2). Successful pDNA encapsulation after jetting and surface‐capping was further confirmed through a dual‐emission fluorescence resonance energy transfer (FRET) study using structured illumination microscopy (SIM, super‐resolution). Alexa Fluor‐647 (AF647) labeled albumin was used for the scPNPs, which were loaded with Cy3‐pDNA and fabricated as explained above (Figure S3). These results confirmed pDNA encapsulation after EHD jetting and after surface‐capping.

A crucial property of successful nonviral gene delivery modalities is the effective encapsulation and stabilization of nucleic acids to protect DNA or mRNA payloads from degradation. The stability of enhanced green fluorescent protein (eGFP)‐encoding pDNA encapsulated in scPNPs was assessed by incubation with DNAse 1, which effectively cleaves accessible pDNA. Agarose gel electrophoresis was then used to compare pDNA scPNPs and naked pDNA (Figure 2B; Figure S4). After exposure to the enzyme, the band associated with naked pDNA disappeared, confirming the effective degradation of the plasmid by DNAse 1 [45]. In contrast, scPNP‐loaded pDNA remained intact, as indicated by the bands at the 3.5 kilobase position. The variation in size relative to the control pDNA may be attributed to a higher propensity of linear and nicked plasmids, rather than supercoiled plasmids [46]. Electrophoretic mobility can sometimes differ if the DNA is kinked, supercoiled, or subject to other conformational changes. Mechanical stress, such as vigorous mixing or shear induced by EHD jetting, may have caused nicked plasmids or relaxed circular plasmids instead of supercoiled plasmids [46], as seen in the scPNP and jetting solution samples. The scPNP control (without DNAse1) displayed the corresponding trends. In total, these results indicate that scPNPs effectively protected the pDNA from enzymatic degradation.

To confirm nanoparticle stability, size and swelling of scPNPs were monitored over time. In water, scPNPs were stable for 12 days with no significant changes in diameter or PDI (Figure 2C). Moreover, the secondary structure of albumin was not measurably impacted by either the jetting or the surface‐capping process based on circular dichroism spectroscopy (Figure S5). We found a high pH responsiveness of scPNPs, a feature that is critical for plasmid release inside of target cells. The pH response of the scPNPs was assessed by studying the changes in the scPNP diameter during exposure to different acidic environments (Figure 2D). A decrease in pH from an initial neutral (pH ∼7) environment resulted in a slight increase in diameter. As seen in Figure 2D, the scPNPs increased in diameter incrementally with protonation of both secondary and tertiary amines of the PEI as the pH dropped from 7.4 to 5.4 [47]. At pH 5.4 through pH 4.5, a notable decrease in size occurred. Eventually, the scPNP average diameter reached a plateau at pH 4.3–4.1. This is further demonstrated by comparing the nDLS at pH ∼7 (original scPNP diameter) to pH ∼4 (disassembled scPNP diameter) in Figure 2E. These results are not surprising given the isoelectric point of albumin (4.7). We attribute the exquisite pH responsiveness to a combination of various effects: (i) At neutral pH, the PEI is confined to the surface of the protein nanoparticles via interfacial complexation while albumin acts as an ampholytic shield separating the DNA from the PEI, (ii) at slightly acidic pH, bPEI is already highly protonated, but continues to protonate as the system is dropped to a pH of 5.4; this increases the positive charge density for PEI and enhances electrostatic repulsion among PEI chains, increasing the osmotic pressure within the protein nanoparticles [48, 49]; we consider this to be similar to the “proton sponge effect,” which has been reported to promote osmotic swelling and structural disruption of nanoparticles [50], and (iii) at even lower pH, conformational changes in zwitterionic albumin can occur due to the successive protonation of amino acid residues; this may weaken noncovalent interactions (hydrogen bonding and hydrophobic interactions) between albumin and DNA, destabilize the nanoparticle matrix, and favor DNA release. We posit that both enhanced PEI protonation (increasing positive charge) and shifts in albumin surface charge (due to protonation of carboxylate groups and reduced electrostatic repulsion) destabilize the balance of forces in the particles, resulting in rapid particle disaggregation and payload release.

Control of loading capacity was demonstrated by formulating scPNPs with increasing pDNA loading from 10% to 40% w_pDNA_/w_albumin_ (Figure 2F–H). No significant differences in diameters and circularities of the as‐jetted PNPs were observed for the different groups. The DNA‐to‐protein ratio can be controlled using EHD jetting without changing major particle properties (Figure 2G). Next, scPNPs with different pDNA loadings were prepared using a constant amount of bPEI (Figure 2H). We observed some variations in size distributions in DLS but there were no differences in the average diameters and the zeta potentials of the scPNPs for different pDNA loadings. The pDNA mass was calculated for each formulation (Supporting Experimental Methods S4) and the average number of plasmid molecules in scPNPs was calculated to be 28 plasmids per average scPNP for the 10% w/w formulation and 99 for the 40% w/w (Supporting Experimental Methods S5). pDNA loadings as high as 70% w/w were studied, but loadings above 40% w/w resulted in jetting instabilities (Figure S6). Genes longer than GFP were also assessed for jetting stability and scPNP fabrication consistency. Size distribution and zeta potentials (Figure S7) for the scPNPs did not exhibit any significant changes when switching between GFP (∼3.5 kb) and luciferase (∼6.8 kb) encoding plasmid DNA.

### 2.3 Optimizing Transfection Efficacy of scPNPs with Varying Loading and Dosage

**FIGURE 3:**
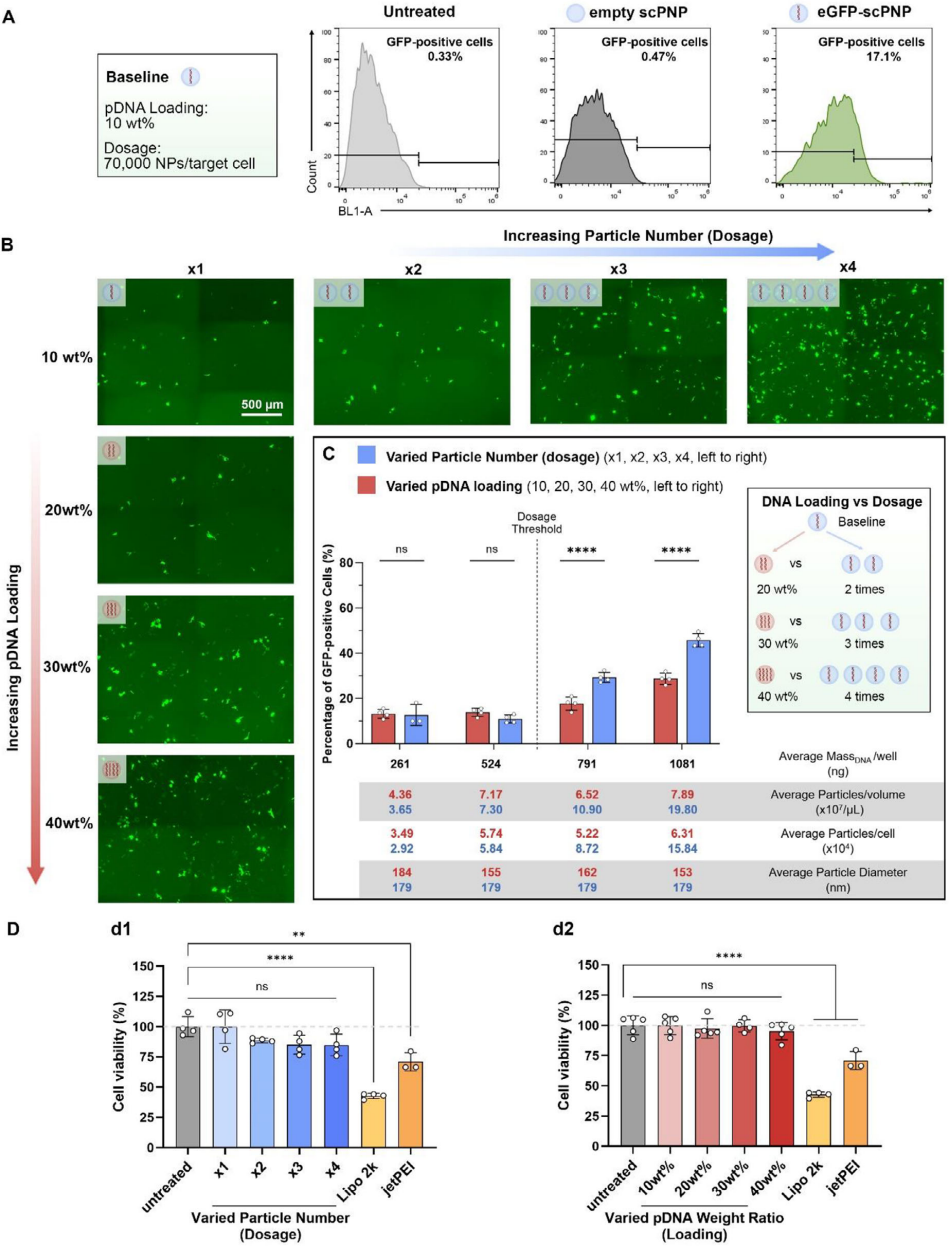
Enhanced and optimized transfection efficacy of scPNPs loaded with eGFP–pDNA: a comprehensive loading and dosage comparison. (A) Transfection efficiency of eGFP–pDNA‐loaded scPNPs with baseline parameters, assessed by quantifying the percentage of HepG2 cells that produce GFP via flow cytometry. (B) Representative live cell images of HepG2 cells, 72 h post‐transfection mediated by eGFP–pDNA‐loaded scPNPs at increased pDNA loading from 10% to 40% wt/wt (red arrow) or increased particle number (dosage), ranging from the baseline dosage to 4 times of the baseline (blue arrow) (*n* = 3, scale bar is 500 µm). (C) Transfection efficiency 72 h post‐transfection (GFP+ % assessed by flow cytometry, mean ± SD, *n* ≥ 3, with each pair of adjacent columns equivalent pDNA mass per well). (D) Cytotoxicity of eGFP–pDNA‐loaded scPNPs measured by WST‐8 in HepG2 cells, 48 h post‐transfection at (d1, blue) varied particle numbers from baseline number to 4 times, and at (d2, red) varied pDNA loadings from 10% to 40% wt/wt. In all instances, significance was determined by two‐way analysis of variance with Turkey's post hoc test (**p < 0.01, ****p < 0.0001).

As a starting point, we used the recommended pDNA dosage from a commercially available transfection reagent (jetPEI, ∼250 ng pDNA per well, 96‐well) to establish a baseline for further investigation of transfection efficacy: (i) standard pDNA loading at 10% wt/wt, as formulated in scPNPs and (ii) standard input dosage at 70 000 (70k) scPNPs per target cell. Based on these baseline parameters, we assessed the transfection efficiency both qualitatively and quantitatively, using eGFP encoded pDNA as the payload. Human hepatocyte‐like cells (HepG2) were incubated with pDNA‐loaded scPNPs for 72 h. The transfection efficiency was evaluated based on the percentage of GFP‐positive cells (i.e., the percentage of live cells with green fluorescence versus the total number of live cells). As a baseline, this initial scPNP formulation successfully transfected HepG2 (∼17% GFP‐positive rate, Figure 3A).

Designing toward enhanced transfection efficiency, we varied pDNA loading and scPNP dosage independent of each other. To decouple the effects of scPNP dosage (particle basis) and pDNA dosage (plasmid basis), two orthogonal paths were taken: (i) increase the pDNA loading while maintaining the scPNP dosage, and (ii) increase the scPNP dosage without changing the pDNA loading. This allowed matching of pDNA mass to compare the transfection efficiency between groups (Figure 3C). Live cell imaging indicated that the transfection efficiency increased gradually with the increase of either gene vector loading or scPNP dosage (Figure 3B). Quantitative analysis via flow cytometry confirmed that the transfection efficiency monotonically, but nonlinearly, increased with the total input pDNA once a certain threshold was reached (Figure 3C). Varying either the pDNA loading or scPNP dosage below the threshold yielded only negligible improvements in transfection efficiency (from 13.2% to 13.8%). However, a pDNA dosage above the threshold concentration of 750 ng/well resulted in significantly improved transfection efficiency. Here, 1000 ng/well (40 wt% pDNA loading or 4 times the baseline scPNP dosage) emerged as the highest performing dosages (up to 50% GFP+).

Higher scPNP dosage, rather than higher pDNA loading, dominated the transfection efficiency trends. In other words, the impact of a particle number increase was more dominant than higher pDNA loading (comparing identical total amounts of plasmid). Higher scPNP dosages correspond to a higher propensity of particle‐endosomal membrane interactions. The increased efficiency of plasmid delivery directly improves endosomal escape of gene vectors, which has been widely recognized as a key rate‐limiting step for nanoparticle drug delivery. Typically only 1%–2% of gene vectors are able to escape vesicular compartments in the limited window of time before various degradation mechanism take effect [51]. Thus, as long as the carriers deliver an adequate number of plasmids (estimated at ∼28 plasmids/scPNP in the 10% w/w scPNP system, detailed in Supporting Experimental Methods), the number of delivered nanoparticles is more important than the plasmid loading when the pDNA masses are equivalent.

Once transfection efficiency was established, we evaluated the cytotoxicity of scPNPs by a WST‐8 test after cells incubated with scPNPs of varying dosages and loadings for 48 h. For all scPNP groups tested in this study, HepG2 cells maintained high cell viability after 48 h of incubation (no significant difference). Specifically, high cell viability (∼85%) was maintained with scPNP dosages up to 4 times the baseline amount in comparison with the untreated group (Figure 3D). This highlights the safety of scPNPs across a broad range of dosages and loadings. These findings favorably compare to even commercial products like jetPEI and Lipofectamine, which typically achieve only about 50% cell viability compared to the control group (Figure 3D) [11].

### 2.4 Rapid and Highly Tunable Cellular Uptake Efficiency of scPNPs

**FIGURE 4:**
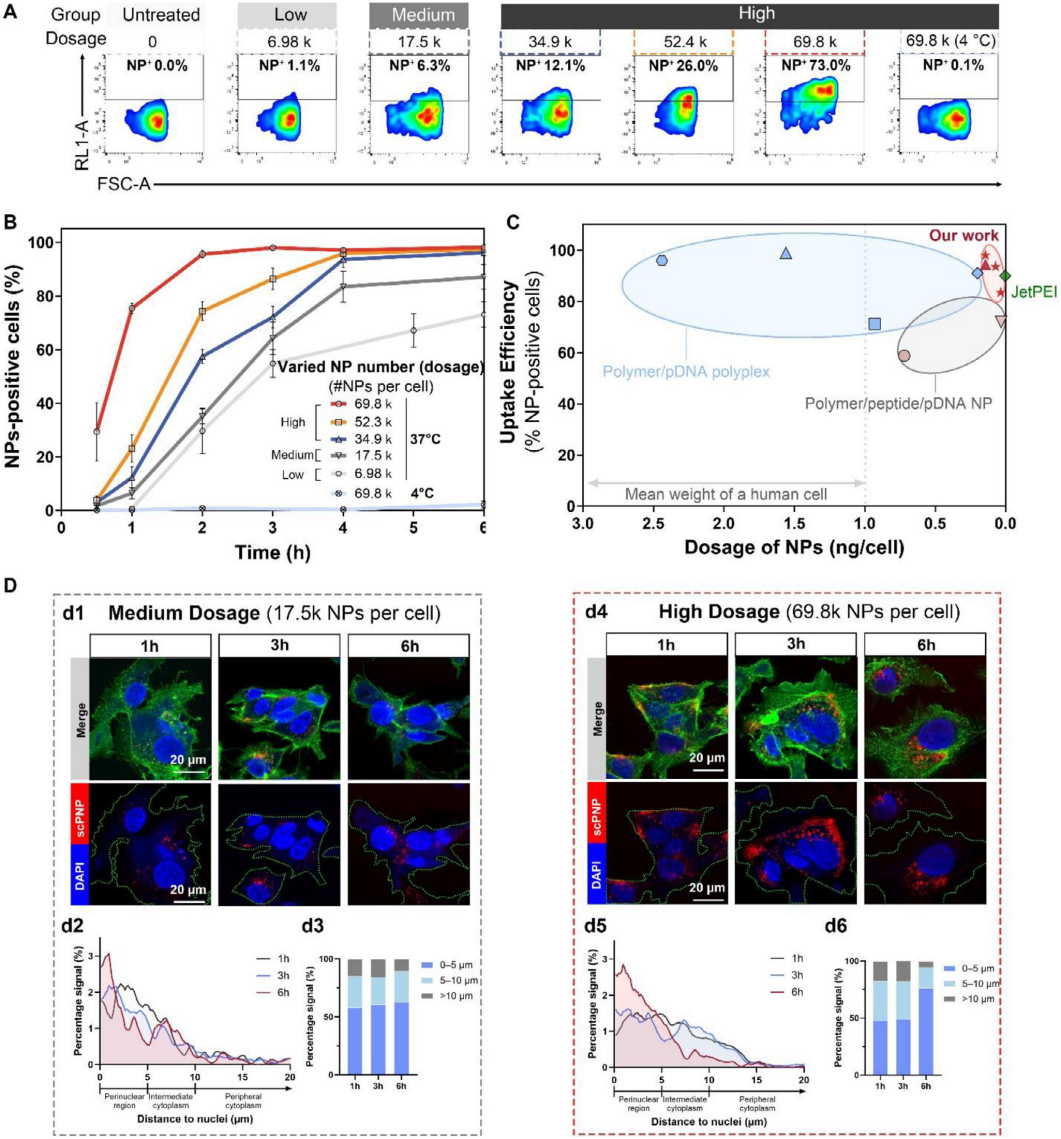
Rapid and highly tunable cellular uptake efficiency of scPNPs. (A) Fluorescence‐based cellular uptake studies of scPNPs in vitro via flow cytometry: representative flow cytometry plots of the HepG2 population taking up AF647‐scPNPs as a function of dosage (scPNP/cell) 1 h post‐incubation; all scPNPs used under this experimental setup were loaded with 10wt% eGFP–pDNA as a model nonviral gene vector. (B) Quantification of the HepG2 taking up with AF647‐scPNP uptake over 6 h, plotted as a function of dosage; data are represented as means ± SD (*n* ≥ 4, biologically independent sample). (C) Comparison of scPNP uptake efficiency in human hepatocyte‐like cells (HepG2, indicated by red asterisk) and human kidney embryonic cells (HEK293T/17, indicated by red triangle) to reported efficiencies in other nanoparticle systems encapsulating pDNA; uptake efficiency, defined as the ratio of nanoparticle‐positive cells to the total cells, was evaluated via flow cytometry 4 h post‐incubation; Details and values of the aforementioned nanoparticle systems are listed in Table S1. (D) Visualization of cellular uptake of AF647‐scPNPs in HepG2 cells by confocal laser scanning microscopy at: (d1–d3) medium dosage, 17.5K scPNP/cell, and (d4–d6) high dosage, 70K scPNP/cell, observed at 1, 3, and 6 h post‐incubation. Confocal imaging channel: Blue—nuclei stained by DAPI; Green—optimized for Alexa Fluor 488 Phalloidin, which allows delineation of cell boundaries (green dotted circles); Red: AF647‐scPNP; Merge: linear combination of the aforementioned channels. Distribution histograms show the percentage of intracellular scPNP signal as a function of distance from the nucleus at 1, 3, and 6 h for medium (d2) and high (d3) dosages. Binned quantification of scPNP accumulation within perinuclear (0–5 µm), intermediate (5–10 µm), and peripheral (>10 µm) regions across timepoints.

We next quantified internalization following the workflow shown in Figure 4A. Cell population fractions exhibiting AF647 fluorescence (% NP‐positive cells, Figure 4A) were assessed as a function of scPNP number (dosage). After a 1 h incubation, >70% of the cell population was NP‐positive (70k per cell, Figure 4A, a2). We also assessed lower dosing levels, including 10%, 25%, 50%, and 75% of the baseline dosage. At the lowest dosage, HepG2 cells exhibited a much slower cellular uptake behavior. Specifically, at 1 h, only 0.76% of cells have taken up scPNPs, corresponding to a ∼100‐fold decrease in absolute scPNP‐positive cell percentage for only a tenfold decrease in the number of scPNPs applied (Figure 4A, a2). Cellular uptake kinetics of scPNP as a function of dosage were measured at 0.5 to 6 h post‐incubation. As shown in Figure 4B, higher input dosages correlated with enhanced uptake rates. Moreover, different uptake kinetics were observed for different dosages. For low and medium doses (7k–17.5k scPNPs per cell), an asymptotic value of 70%–80% scPNP‐positive cells was reached by hour 6. However, for high doses (35k–70k scPNPs per cell), almost every cell eventually internalized scPNPs, with slower or more rapid uptake for lower or higher scPNP doses. We speculate that concentrations above the threshold dosage can overwhelm the cellular uptake capacity through receptor and binding site saturation [52]. Although the specific dosage values may vary between different nanoparticle designs (surface potential, size, etc.), it points toward the possibility of a foundational dosage threshold for nanoparticle delivery, particularly for protein‐based nanoparticles.

Further investigations using confocal microscopy (1, 3, 6, 48, and 72 h post‐incubation) revealed a medium dose of scPNPs sparsely distributed through the cytoplasm without any clear progression into the intracellular trafficking pathways (Figure 4D, d1, 17.5k scPNP/cell). In contrast, a high dosage (70k scPNP/cell) resulted in regional clustering within the cytoplasm. scPNPs are initially observed close to the cell membrane at 1 h. They then gradually progress into perinuclear regions by 3 and 6 h (Figure 4D, d2). At 48 and 72 h, scPNPs accumulate near the nucleus and produce cytosolic/nuclear GFP with a high dose of scPNPs, which persists through 72 h (Figure S8). We propose that applying high doses to cells overwhelms the intracellular trafficking pathways, thus leading to a multiphased pattern of scPNP distribution in the cytosol (e.g., cell membrane → nucleus). It has been reported that maturing endosomes move from the cell periphery to the center of the cells [53], suggesting that endosomes closer to the nucleus are more likely to be acidified late endosomes or lysosomes [54]. Therefore, the trafficking of scPNPs to the perinuclear region provides an increased possibility of endosomal escape when in close proximity to the nucleus. This not only provides a shorter distance for the plasmid to diffuse to the nucleus [44], but also provides the right window of time for dissociation and escape. Hence, it is argued here that the nuclear proximity of endosomal escape in the high dose regime has an effect on the transfection efficiency.

It is worth noting that at 4 h post‐incubation, more than 93% of the cells are AF647‐scPNP positive under high dose conditions (Figure 4C). This uptake rate outperforms several well‐established nanoparticle systems, including jetPEI on a pDNA mass basis (Table S1). Payload encapsulation efficiency is a critical attribute for both the efficacy and toxicity of nanoformulations in human use [55]. However, the most commonly used classes of nanoparticles, including LNPs and polymer‐based nanoparticle systems, are still limited by low drug loading [3]. On an encapsulation basis (carrier‐to‐pDNA), our scPNP system exhibits extremely high loading efficiency, particularly in contrast to other protein‐based or polymer‐based pDNA delivery systems, such as peptide/pDNA complexes or polymer/pDNA polyplexes.

### 2.5 scPNPs Engage Multiple Endocytosis Pathways Contributing to the Enhanced Transfection and Cellular Uptake

**FIGURE 5:**
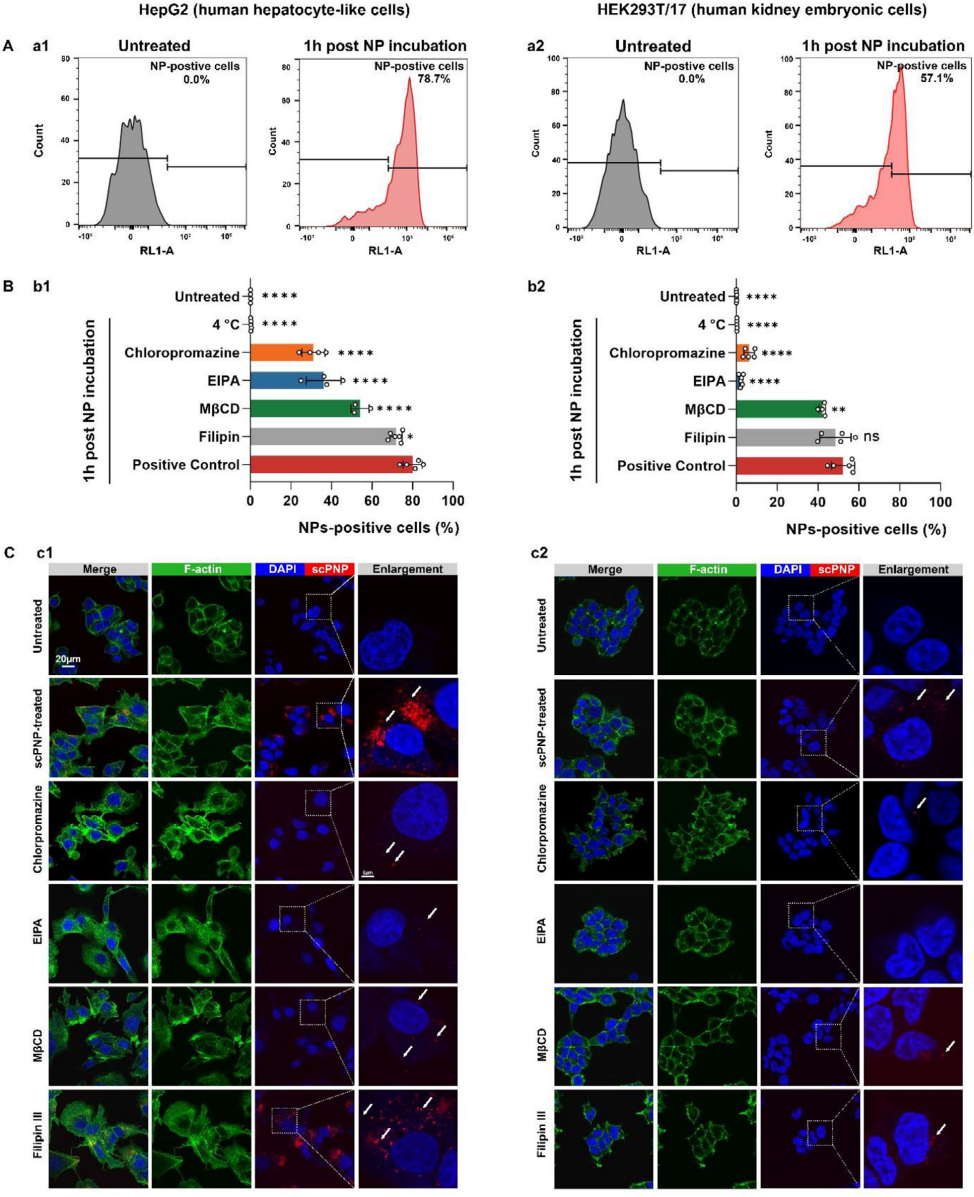
Multiple endocytosis pathways contribute to enhanced transfection and cellular uptake of scPNPs. (A) Flow cytometry histograms demonstrating cellular uptake of untreated and AF647‐scPNP in (a1) HepG2 cells and (a2) HEK293T/17 cells for 1 h. AF647 (RL1)‐A signifies fluorescence intensity within the cells. (B) Effect of temperature and endocytosis inhibitors on cellular uptake of the AF647‐scPNP in different cell lines (b1: HepG2 cells; b2: HEK293T/17 cells). For reference, the noninhibited internalization of the AF647‐scPNP at 1 h was used as a positive control. Data are represented as means ± SD (*n* ≥ 4, biologically independent sample). One‐way analysis of variance, with post hoc Dunnett's multiple comparisons, was used to determine significance (**p < 0.01; ****p < 0.0001) in comparison to the control. (C) Confocal laser scanning microscopy images of AF647‐scPNP in (c1) HepG2 cells and (c2) HEK293T/17 cells after a 1 h incubation in the presence of different endocytosis inhibitors. Green channel, Alexa Fluor 488 Phalloidin, a high‐affinity F‐actin probe; blue channel, nuclei stained by DAPI; red channel: AF647‐scPNP; merge, combination of the channels aforementioned channels. Scale bars: 20 µm in normal magnification and 5 µm in enlarged view. White arrows point to representative examples of AF647‐scPNP.

The cell entry mechanism of scPNPs is not yet understood. We thus blocked specific cell uptake pathways to dissect the endocytic mechanisms contributing to the very high transfection and uptake rates. Chlorpromazine, a clathrin‐mediated endocytosis (CME) inhibitor, largely suppressed the cellular uptake of AF647‐labeled scPNPs in HepG2 cells (>60% reduction). Meanwhile, 5‐(N‐ethyl‐N‐isopropyl) amiloride (EIPA), an inhibitor of macropinocytosis, significantly reduced scPNP internalization. In contrast, Filipin III, a caveolae‐mediated endocytosis inhibitor, produced only minor inhibition (∼10% decrease, *p* = 0.03). Inhibition of either CME or macropinocytosis reduced uptake by 50–60%, suggesting that both pathways are required. At low temperature (4°C), the scPNPs were internalized in less than 0.3% of cells after 1 h incubation. A low temperature decreases the metabolism of cells (Figure 5B), in contrast to cellular uptake of ca. 80% at 37°C (Figure 5A). All of these results suggest that scPNPs can rapidly (≤1 h) undergo efficient, energy‐dependent cellular uptake that is mediated by multiple endocytosis pathways, primarily by macropinocytosis and CME. A control experiment, using non‐AF647 labeled scPNPs vs. labeled scPNPs was performed to evaluate whether the cellular uptake is due to the fluorescently labeled particles and not because of nanoparticle scattering effects (Figure S9).

Notably, HEK293T/17 cells, a type of human kidney embryonic cells, exhibited a similar trend under the same pharmacological inhibition conditions. The HEK293T/17 model demonstrated a higher dependency on CME and macropinocytosis, almost entirely inhibiting cellular uptake (Figure 5B, b2). This difference in endocytosis mechanisms may be attributed to two factors. First, there is an absence of caveolae in HEK293T/17 cells, mainly due to the lack of cavin1. Second, the size of the scPNPs (∼177 nm) may play a role. This size makes them ideal for classical CME (∼100 nm) [56] and macropinocytosis (>200 nm), but not for caveolae‐mediated endocytosis (<60 nm). Interestingly, despite methyl‐β‐cyclodextrin (MβCD) being commonly used as a caveolae inhibitor, it still significantly reduced the cellular uptake in both cell types (∼30% decrease in HepG2; ∼20% decrease in HEK293T/17). This is because MβCD is different from inhibitors with high specificity like chlorpromazine, EIPA, and filipin III. These differences may interfere with other uptake mechanisms due to changes in membrane fluidity [57]. It is important to recognize that many endocytosis inhibitors work on multiple pathways [58], which makes it difficult to draw definitive conclusions about a singular endocytic pathway due to a confluence of mechanisms. In this study, we selected the inhibitors with highest specificity and optimized the concentration of inhibitors to ensure minimized side effects in cytotoxicity [59].

Confocal microscopy was used to further monitor cellular uptake, scPNP spatial distribution, and accumulation of scPNPs under different pharmacological treatments. Based on images collected after 1 h incubation (Figure 5C), a significant inhibition of scPNP internalization can be clearly observed in chlorpromazine‐ and EIPA‐treated groups for both cell types, which is consistent with flow cytometry data (Figure 5B). The scPNP accumulation patterns, revealed in enlarged confocal images, exhibited considerable variations in the two different cell types. The AF647‐scPNPs delivered to human kidney embryonic cells (HEK293T/17) were very limited in number and dispersed in the cytoplasm, while scPNPs clustering could be observed in the cytoplasm of HepG2. Previous study of LNPs, with similar endocytosis pathways to scPNPs [51], has also demonstrated cell‐type specific differences in internalization processes. These findings suggest that scPNPs enter cells in a cell type‐specific manner, and the cell specificity is very likely to be correlated to protein expression(s) related to intracellular trafficking of scPNPs [51, 60].

These results raise the following question: What specific features of scPNPs are responsible for enhanced transfection and cellular uptake? For a typical transfection‐effective system of bPEI‐DNA polyplexes (the molar ratio of nitrogen from PEI to phosphate from DNA (N:P) reaches ∼10), only ∼30% of bPEI polymers are required to condense anionic pDNA chains. Typically, ∼70% of added bPEI binding sites are not associated with a nucleic acid phosphate [10, 61]. The fabrication methods employed in this work avoid direct contact between the plasmid and PEI. In fact, it has been previously demonstrated that interfacial bPEI chains (molar mass of 25 000 g/mol) can: (i) reduce lysosomal entrapment of internalized PEI‐DNA polyplexes, (ii) assist in translocation of pDNA through nuclear membranes after it is released from polyplexes in the cytosol, (iii) enhance pDNA‐to‐mRNA transcription efficiency, and (iv) facilitate nucleus‐to‐cytosol translocation of mRNA [61]. Given that excess cationic moieties promote gene transfection in each step listed above, we propose that the excessive free bPEI cations play an important role in enhancing the transfection efficiency of scPNPs.

Unbound cationic chains and oligomers have been shown to increase the uptake rate, with major uptake on time scales between 0.5–1.5 h. This is in good accordance with the sharp increase in the scPNP‐positive ratio observed in this experiment, also suggesting that excess of PEI cationic moieties at the scPNP surface may be very important to cellular uptake in the first 1.5 h. However, the mechanism of these cation‐facilitated internalization enhancements has yet to be fully elucidated. Based on the cytotoxicity outcomes presented here (Figure 3D), free polycations promoting cellular internalization by destabilizing the cell membrane can be ruled out. Had this been the case, one would have expected a PEI concentration dependence or scPNP concentration dependence in the toxicological results, which was not observed.

### 2.6 Transfection, Cellular Uptake, and Cytotoxicity of Human Primary T‐Cells

**FIGURE 6:**
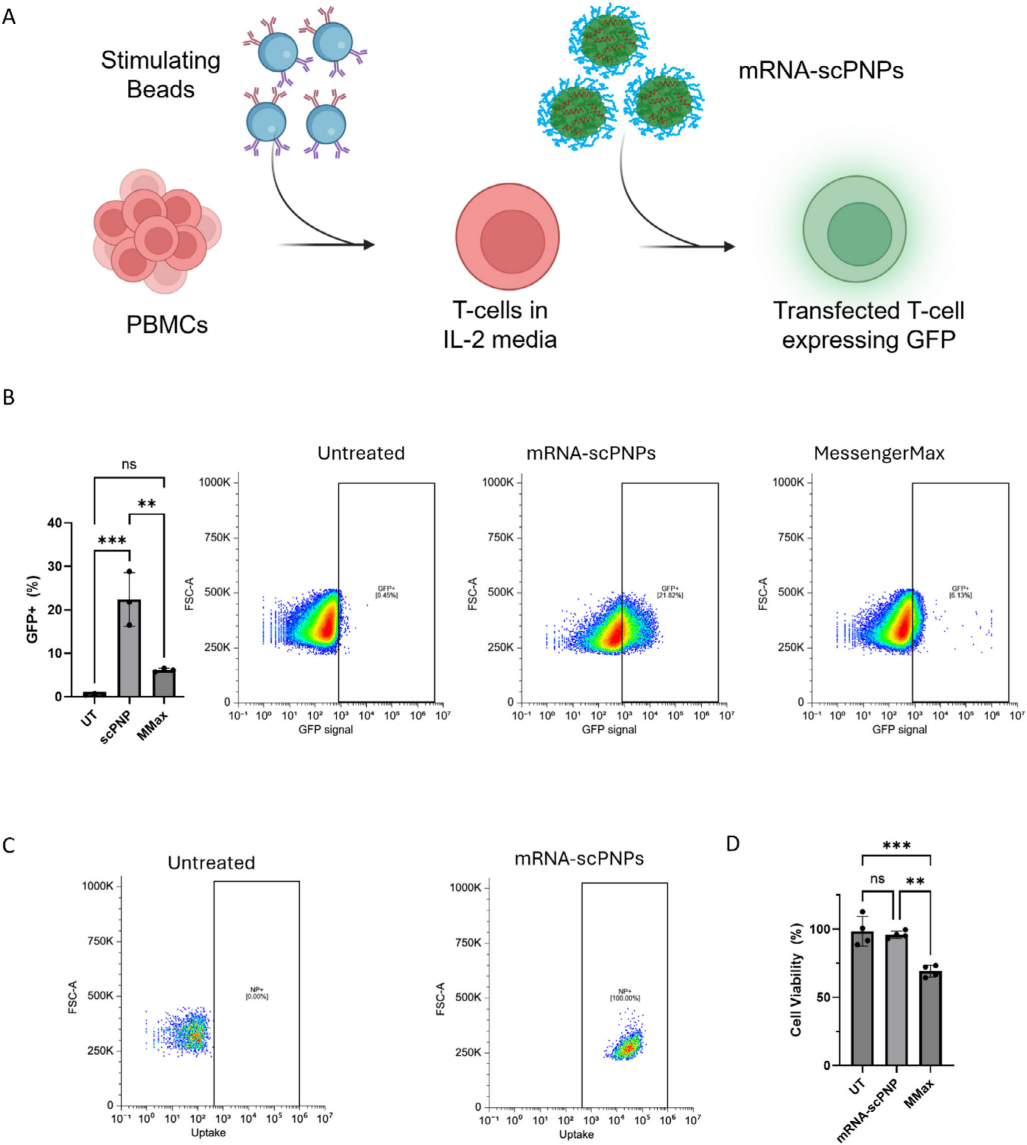
Transfection, cellular uptake, and cytotoxicity of scPNPs treated human primary T‐cells. (A) Schematic of the experimental procedure. (B) Transfection efficacy of T‐cells treated with mRNA‐loaded scPNPs and Lipofectamine MessengerMax after 48 h of incubation, expressed as % GFP positive cells. (C) Cellular uptake of T‐cells treated with AF647‐labeled scPNPs, after 24 h of incubation. (D) Cell viability of T‐cells treated with mRNA‐loaded scPNPs and MessengerMax after 48 h of incubation. Bars represent mean ± SD (*n* = 3 biologically independent samples).

To further emphasize the translational potential of our scPNPs, we expanded our experimental scope to include delivery to ex vivo blood cells, focusing on mRNA delivery in primary human T cells. T cells are central to CAR‐T manufacturing; however, they are consistently difficult to transfect via nonviral methods. Primary T cells were isolated from primary human peripheral blood mononuclear cells (PBMCs) and expanded in IL‐2 containing medium to promote cell growth and survival, followed by treatment with mRNA‐loaded (30% w/w) scPNPs (Figure 6A). mRNA–scPNP physical and surface properties were similar to those of pDNA–scPNPs (Figure S10).

25% of cells were transfected (GFP+) after 48 h of incubation with mRNA‐loaded scPNPs (Figure 6B). This was fourfold higher than Lipofectamine MessengerMax (∼6% GFP+ cells) at the same mRNA dosage (∼400 ng/well). Primary human T cells are well known for their resistance to nanoparticle uptake, making effective delivery challenging [14, 15]. Nevertheless, rapid particle internalization (>99% positive cells) was evident within 24 h of incubation with AF647‐labeled mRNA–scPNPs (Figure 6C), similar to the pDNA–scPNP uptake profiles in adherent cell lines above. Importantly, T cell viability was maintained at 96 ± 2% with scPNP treatment, in contrast to Lipofectamine MessengerMax, which reduced viability to 69 ± 3% (Figure 6D). This is particularly notable given that high viability is mandatory for CAR‐T manufacturing [62], and cytotoxicity is one of the main limitations of current transfection methods such as electroporation.

## 3 Conclusion

In pursuit of developing a protein‐based nanoparticle platform for highly efficient delivery of large‐sized nucleic acids, this study introduces scPNPs as efficient nonviral gene carriers. scPNPs exhibit pH‐responsiveness, a wide range of pDNA and mRNA loading fractions w/w without changing the scPNP properties, and high production yields. Above a threshold of 750 ng/well pDNA, increasing scPNP dosage has a higher impact on the transfection efficiency than increasing pDNA loading when the total plasmid number is held constant. Additionally, the internalization efficiency is readily controlled by simply tuning scPNP dosage without the need for reformulating scPNPs. Importantly, our findings highlight that this platform achieves robust gene transfer via mRNA delivery in primary human T cells while maintaining high cell viability, surpassing Lipofectamine MessengerMax. Our experiments further demonstrate the versatility of tuning scPNP design parameters, including gene vector size, choice of polycations, and protein carriers. Future uses of the scPNP platform may include a variety of payloads (e.g., siRNA, antisense oligonucleotides, and Cas9‐sgRNA complexes) for expansion into a multitude of therapeutic areas. Further efforts are still needed to thoroughly understand the mechanism leading to the very rapid internalization of scPNPs. This work established a new platform of high‐density nucleic acid delivery systems, featuring high nucleotide‐to‐carrier ratios and effective particle sizes between 100–200 nm, which will open novel avenues for nanoparticle‐based applications in medicine, ultimately augmenting or even replacing delivery via microinjection or electroporation.

## 4 Experimental Section

### 4.1 Materials

pCMV‐GFP (#PF463) was purchased from Plasmid Factory. Ultrapure albumin from bovine serum (BSA, #AM2616), Alexa Fluor 647 conjugated BSA (#A34785), Pierce 660 protein assay (#1861426), Tris Acetate EDTA (ethylenediaminetetraacetic acid) buffer (#B49), SYBR safe gel stain (#533102), and DNAse1 kit (#EN0521) were purchased from Thermo Fisher Scientific. Branched polyethyleneimine (#408727), methanol (#179337), sodium hydroxide (#S9045), acetic acid (#A6283), and heparin sodium salt (#H4784) were purchased from Sigma Aldrich. Lacey‐carbon TEM (#01824, Ted Pella), agarose (#A201100, GoldBio), gel loading dye, purple (#B7024S, BioLabs) were also used.

### 4.2 Nanoparticle Fabrication

scPNPs were prepared using EHD jetting [24, 25, 26, 39] followed by a surface stabilization step with bPEI. Briefly, the jetting solution consisted of BSA and pDNA encoding for eGFP (3487 bp), in a 7/3 ultrapure (UP) distilled water (RNAse, DNAse free) and methanol solvent. The final concentration of BSA was kept constant at 0.1% w/v and the pDNA concentration varied from 10% to 40% w/w of BSA. BSA was dissolved and sonicated after mixing with the water and methanol solvent via bath sonication prior to adding the pDNA. The solution was pumped at 0.06 mL/h through a 25‐gauge blunt tip needle and a voltage of ∼6 kV was applied to produce a stable Taylor cone. The applied voltage caused the formation of nanodroplets, which form solid nanoparticles once the solvent evaporates. The distance from the tip of the needle to the collecting surface was maintained at 6 cm.

### 4.3 Nanoparticle Collection

Immediately after jetting, a dilute solution of bPEI (∼8 µg/mL) was added to the collecting pans containing protein nanoparticles, to produce scPNPs. The scPNP solution was sonicated via a horn sonicator at 50 A for 30 s. To remove free PEI and reconcentrate the scPNP solution, the samples were centrifuged at ∼18 000 g for 30 min at 10°C and the pellets were resuspended in the desired solvent.

### 4.4 SEM

During jetting, silicon wafers were placed on the collection pans and used for imaging via SEM (Thermo Fisher Nova 200 Nanolab Dualbeam FIB) at the Michigan Center for Materials Characterization. For imaging scPNPs, a buffer exchange was performed using 100 kDa centrifuge filters to remove any free molecules from the scPNP solution. A small volume of solution was drop‐casted onto silicon wafers and dried in a desiccator. Prior to imaging, SEM samples were sputter‐coated with gold for 30 s using SPI‐module Carbon/Gold Sputter Coater for monolayer deposition. Immersion field view was used, and measurements were taken at 5 kV acceleration voltage and a current of 0.40 nA. The images were analyzed via ImageJ for size and shape characterizations in their dry state. It was important to note that the particle density on the SEM chip did not influence the size distribution analysis, as shown in Figure S11.

### 4.5 TEM and EDS

TEM imaging and EDS were obtained on a TFS Talos TEM (F200X G2 S/TEM) equipped with a Super‐X EDS detector at the Michigan Center for Materials Characterization. 300‐mesh size TEM grids with a lacey carbon support film were placed on the collection pan while jetting. Images were captured with STEM mode with an accelerating voltage of 300 kV. EDS scans were ∼20 min long with a dwell time of 10 µs.

### 4.6 DLS and Electrophoretic Light Scattering (ELS)

The hydrodynamic diameter distribution and the zeta potential were acquired using a Zetasizer Nano ZS (Malvern ZSP ZEN‐5600) after collection and suspension in bPEI solution. Particles were sonicated in an ice bath prior to DLS measurement. The reported results on diameter distributions are averages of three measurements.

### 4.7 Circular Dichroism Spectroscopy

scPNPs were diluted in UP H_2_O to match the concentration of the BSA protein solution. BSA and scPNP solutions were characterized in a 1 cm path length quartz cuvette with a Jasco J‐815 CD spectrophotometer. The instrument was equilibrated in nitrogen at 25°C for 30 min before measurement. Measurements were taken in 1 nm increments, with 4 s averaging time and 50 nm/min scan speed.

### 4.8 Gel Electrophoresis

To confirm the DNA encapsulation and stability within scPNPs, a gel electrophoresis assay was performed using 1% w/v agarose gel and a 1X Tris‐Acetate EDTA (TAE) buffer. For enzyme stability testing, the entire pDNA–scPNP solution was subjected to pretreatment before loading onto the gel. scPNPs were incubated with DNAse1 for 10 min at 37°C. To inactivate the enzyme, samples were incubated at 65°C for 10 min. The pDNA release from the scPNPs was made possible by the addition of heparin, an anionic competitor, to replace the pDNA. This, in turn, triggered scPNP disassembly and pDNA exposure to the gel [31, 63]. A heparin solution in 0.1 m NaOH was used at a concentration of 600 ng/1 ng of pDNA. Control pDNA and scPNPs (without DNAse1) were exposed to the same heating and heparin conditions as the samples incubated with DNAse1. After dissociation, samples and controls were mixed with a purple gel loading dye and the resulting solution was loaded onto the gel, where each well contained ∼200 ng pDNA. After gel electrophoresis for 1 h at 120 V, the gel was imaged using FluorChem M Imaging System with the chemiluminescence blue laser with a green filter (537 nm).

### 4.9 pH Measurements

To study the effect of pH on the scPNP stability, 2.5% v/v acetic acid was added to a scPNP solution in increments of 0.1 µL, and the samples were allowed to reach equilibrium (∼2 min) after each addition. Diameters were measured via DLS. Similarly, the pH values of the solution were measured after each addition using a Mettler Toledo pH meter.

### 4.10 Cell Culture

The human hepatocyte‐like cell line, HepG2 [ATCC HB‐8065] was purchased from ATCC (USA) and was maintained using Eagle's Minimum Essential Medium (EMEM), supplemented with 10% heat‐inactivated fetal bovine serum (FBS) and 100 U/mL Penicillin/100 µg/mL Streptomycin (Invitrogen). The human kidney embryonic cell line, HEK293T/17 [ATCC CRL‐11268] was purchased from ATCC (USA) and was maintained in Dulbecco's Modified Eagle Medium (DMEM) supplemented with 10% heat‐inactivated FBS and 100 U/mL Penicillin/100 µg/mL Streptomycin. Both cell lines were incubated at 37°C and 5% CO2 until around 90% confluency for the cell seeding.

### 4.11 Transfection

To reach a monolayer of cell growth, prior to cell seeding, rat tail collagen coating solution (Sigma‐Aldrich) was added to each well of 96‐well cell culture plate (Corning) with a culture surface ratio of 5 µg/cm^2^, based on manufacturer's protocol. After 2 h of incubation at 37°C, collagen coating solution was aspirated gently, followed by rinsing the coated surface with phosphate buffered saline (PBS, Invitrogen). HepG2 cells were then immediately seeded at a density of 1 × 10^4^ per well in the coated 96‐well cell culture plate, 24 h before transfection to reach 60–80% confluence. After 24 h of cell growth, following the removal of medium, 150 µL of fresh 10% serum‐containing EMEM was added per well, and 40 µL of scPNP solution of varying pDNA loadings or particle dosages (corresponding to ∼250, 500, 750, or 1000 ng input pDNA per well) were subsequently added in replicates of 4 or more. Of note, in order to eliminate the interference of auto‐fluorescence of fluorescent dyes under the GFP channel, scPNPs were not labelled with any type of fluorescent dyes for the transfection study. To visualize eGFP–pDNA‐transfected cells, EVOS M7000 Imaging System (Thermo Fisher) was used to observe live cells under both the GFP channel and the bright field over time (24, 48, 72 h). Specifically, cells were observed on a larger scale by the EVOS M7000 Imaging System under the magnification of 10× to capture an image at the scale of 2500 × 1875 µm by merging four single images together (Figures S12 and S13).

### 4.12 Quantifying Transfection Efficiency through Flow Cytometry

At 72 h post‐scPNP‐mediated transfection, the cells were washed with PBS and detached from the 96‐well cell culture plate surface by 0.25% trypsin‐EDTA (Gibco) for 2 min. The cell suspension collected was diluted with ice‐cold PBS containing 1% FBS and centrifuged at 4°C at 500 g for 5 min in 15 mL conical centrifuge tubes. The supernatant was then discarded, and the cell pellets were resuspended in ice‐cold PBS and used for running the flow cytometry (Attune Flow Cytometer, Applied Biosystems, Figure S12). Untreated cells were used as negative controls for fluorescence gating. The BL‐1 (488 nm) laser was used for detecting eGFP‐positive cells. Flow cytometry data were analyzed using FlowJo (FlowJo LLC).

### 4.13 Quantifying Cell Viability by CCK‐8 Test

Cell viability was measured using the colorimetric assay with CCK‐8 (Dojindo). 48 h after scPNP‐mediated transfection, the cells were treated with 10% solution of CCK‐8 in the serum‐free EMEM and incubated at 37°C with 5% CO_2_ for 1 h. After 1 h incubation, the supernatant was transferred to a clear‐bottom black 96‐well plate (Corning), and the absorbance of the supernatant at 460 nm was measured by the plate reader (SpectraMax M5e, Molecular Devices). Absorbance from 10% CCK‐8 solution in pure EMEM was subtracted from all data points, and the values were normalized to the absorbance from the supernatant of untreated cells. Negative controls were performed with cells being similarly treated with media that was equivalently diluted by ultrapure sterile water but did not contain any particles.

### 4.14 Quantifying Cellular Uptake through Flow Cytometry

Cells were transfected according to the typical protocol using the AF647‐labelled scPNPs (Figure S14). At time points of 0.5, 1, 2, 3, 4, and 6 h after scPNP incubation, the cells were washed with PBS and incubated with 50 µL of CellScrub Buffer (Amsbio) for 10 min at room temperature (RT), which is designed to remove all complexes of DNA and cationic lipids that associate with cell surfaces during transfection. After the incubation of CellScrub Buffer, cells were washed with PBS one more time and detached from the cell culture plate using 0.25% trypsin‐EDTA for 2 min. The cell suspension was diluted with ice‐cold PBS containing 1% FBS and centrifuged at 4°C at 500 g for 5 min in 15 mL conical centrifuge tubes. The supernatant was discarded, and the cells were resuspended in ice‐cold PBS and used for flow cytometry measurements. RL‐1 (637 nm) laser was used for detecting NP‐positive cells.

### 4.15 Confocal Microscopy

The general transfection protocol was followed but in a chamber slide‐based system for collecting high‐quality images from the confocal microscopy. Eight‐well chambered slides (Thermo Fisher) were coated with collagen, followed by seeding with HepG2 or HEK293T/17 cells with a seeding density of 22 000 cells per well. At time points 1, 3, and 6 h after transfection, the cells were washed gently with PBS followed by the incubation of CellScrub Buffer for 10 min. After the CellScrub treatment, cells were fixed with 4% paraformaldehyde for 15 min at RT. After fixation, the fixative solution was discarded, and the cells were washed gently with PBS 3 times, followed by permeabilization with 0.25% Triton‐X100 (in PBS) for 10 min. After the permeabilization solution was discarded, and the cells were washed 3 times with PBS, Alexa Fluor 488 Phalloidin (1:400, Invitrogen) solution was added to each well to stain the cytoskeleton. To visualize the cell nuclei, scaffolds were stained with DAPI staining solution (1:1000, Abcam) for 10 min at RT. After that, the residual buffer was removed carefully, and chamber slides were mounted using SlowFade Diamond Antifade Mountant (Thermo Fisher), and the cells were imaged on the same day. All images were collected on Nikon A1si confocal microscopy system, equipped with a 40X water objective (NA 1.70). Laser illumination consisted of 405, 488, and 640 nm lasers were turned on to detect cell nuclei, cell cytoskeleton, and AF647‐scPNPs, respectively.

### 4.16 Confocal Microscopy Image Analysis

To quantitatively assess the intracellular location of scPNPs, a custom, high‐throughput image analysis workflow was implemented in Fiji to measure the distance of each particle‐positive pixel from the nearest nucleus across multiple fields of view. Nuclear and particle regions were first identified and segmented in confocal microscopy images under the same magnification and resolution. After the segmentation, a Euclidean distance map was generated to calculate the spatial relationship between particles and nuclei. The raw distance histograms were processed to allow for accurate comparison between different experimental conditions. For final analysis, these histograms were cleaned by removing artifacts. Pixel distances were converted to absolute distances using a scaling factor of 1 pixel = 0.15537 µm. The resulting distance distributions were compared in order to assess dose‐dependent differences in intracellular distribution.

### 4.17 Pharmacological Inhibitors

To identify possible internalization mechanism of scPNPs, following the general transfection protocol, after 24 h of cell growth, HepG2 cells and HEK293T/17 cells were preincubated at 4°C or treated with specific inhibitors at 37°C for 30 min, then incubated with the baseline input dosage of scPNPs at 4°C or in the presence of inhibitors at 37°C for another 1 h. The concentration of inhibitors was: (i) 10 µg/mL chlorpromazine; (ii) 50 µm EIPA; (iii) 1.5 mm MβCD; (iv) 1 µg/mL filipin. All of the inhibitor concentrations are within the reported optimal range and pre‐tested in our lab to ensure minimal toxicity. The cellular uptake of scPNP with the baseline input dosage without any treatments was used as the positive control. The results were shown as relative uptake rate of different groups which were normalized according to the positive control.

### 4.18 T‐Cell Expansion and Transfection

Human PBMCs (STEMCELL Technologies, cat #70025.1) were cultured in RPMI‐1640 medium (Gibco, cat#61870‐036) containing 10% FBS (Gibco, A5670801) and 1% penicillin‐streptomycin (Gibco, cat# 15140122). For expansion, cells were plated at 1 × 10^6^/mL and stimulated with anti‐CD3/CD28 magnetic beads (Biolegend, 422603) at a bead‐to‐cell ratio of 1:1 for 10 days. Purity was confirmed with flow cytometry using anti‐CD3‐PE (clone OKT3, Biolegend, cat #B414842) and DAPI (Thermo Fisher cat D21490) viability stain. CD3/CD28 beads were removed and cells were frozen and thawed for transfections. For transfection, human T‐cells were grown in complete RPMI in the presence of 500U/mL recombinant human IL‐2 (Peprotech IU/mL, cat #200‐02‐10UG). Cells were maintained at 37°C, 5% CO_2_, and fed every 2–3 days with fresh medium supplemented with IL‐2. Cells were at 1 × 10^6^ cells/mL at the time of transfection. The cells were seeded at 1 × 10^5^ cells per well in a 96‐well plate and incubated with scPNPs and control groups for 48 h. The amount of mRNA in each well was ∼400 ng for both scPNP and Lipofectamine MessengerMax groups. Untreated cells were used as control for the flow experiments.

### 4.19 Statistical Analysis

All the experiments were performed in three or more replicates and data are represented as mean ± SD unless otherwise indicated. For statistical analyses, Prism 10 software (GraphPad Software, CA, USA) was used. Results were analyzed using one‐way analysis of variance (ANOVA) with Dunnett's posttests or two‐way ANOVA with Sidak's multiple comparison test to examine differences between different groups. Significance levels: *p < 0.05, **p < 0.01, ***p < 0.001, ****p < 0.0001.

## Funding

This study was supported by NIH R01NS124167 (J.L.) and NIH T32CA009676 (F.X.).

## Conflicts of Interest

The authors declare no conflicts of interest.

## Supporting information




**Supporting File**: adma72657‐sup‐0001‐SuppMat.docx.

## Data Availability

The data that support the findings of this study are available from the corresponding author upon reasonable request.
