# Supplementary material for: Surface‐Capped Protein Nanoparticles for Nonviral Gene Delivery
Source: Adv Mater. 2026 Mar 12;38(20):e21796. doi: 10.1002/adma.202521796 (PMC13054114; doi:10.1002/adma.202521796)
Supplement: Supplementary file 1 — Supporting File: adma72657‐sup‐0001‐SuppMat.docx. [file ADMA-38-e21796-s001.docx]

**Supplementary Information**

**Surface-Capped Protein Nanoparticles for Non-Viral Gene Delivery**

Fjorela Xhyliu^1,2,7^, Yao Yao^1,3,7^, Yeongun Ko^1,2,4,7^, Grant Grasman^1,2^, Jeffery E. Raymond^1,2^, Albert Chang^1,5^, Yuxuan Deng^1,5^, Grant Dominic^1^, Michael Triebwasser^1,6^, and Joerg Lahann^1,2,5*^

Address:

^1^ Biointerfaces Institute, University of Michigan, Ann Arbor, MI 48109, USA;

^2^ Department of Chemical Engineering, University of Michigan, Ann Arbor, MI 48109, USA;

^3^ School of Dentistry, University of Michigan, Ann Arbor, MI 48109, USA;

^4^ School of Polymer Science and Engineering, Chonnam National University, Buk-gu, Gwangju 61186, South Korea;

^5^ Department of Material Science and Engineering, University of Michigan, Ann Arbor, MI 48109, USA;

^6^ Division of Pediatric Hematology and Oncology, Department of Pediatrics, University of Michigan, Ann Arbor, MI 48109, USA;

^7^ F.Xhyliu, Y.Yao, and Y.Ko contributed equally to this work.

***Corresponding Author:**

Joerg Lahann

Department of Chemical Engineering, University of Michigan

2800 Plymouth Road, Ann Arbor, MI 48109

Email: [lahann@umich.edu](mailto:lahann@umich.edu)

Supporting Figure 1: Yield analysis on scPNPs loaded with Cyanine-3 (Cy3)-labeled eGFP-pDNA

Supporting Figure 2: Energy dispersive spectroscopy of pDNA-loaded (10% w_pDNA_/w_albumin)_ as-jetted PNPs

Supporting Figure 3: Dual emission FRET study of AF647-labeled scPNPs loaded with Cy3-labeled eGFP-pDNA via structure illuminated microscopy (SIM, super-resolution)

Supporting Figure 4: Gel electrophoresis assay of scPNPs and formulation mixture with and without incubation with DNAse1

Supporting Figure 5: CD spectroscopy of scPNPs and control groups

Supporting Figure 6: Characterization of 70% (w_pDNA_/w_albumin_) eGFP-pDNA loaded scPNPs

Supporting Figure 7: Characterization of scPNPs encapsulating Luciferase-encoding plasmid

Supporting Figure 8: Confocal images of HepG2 cells treated with high and medium dosages of scPNPs at 48 h and 72 h

Supporting Figure 9: Cellular uptake of HepG2 and HEK293T cells incubated with AF647-labeled and unlabeled scPNPs

Supporting Figure 10: Characterization of mRNA-loaded scPNPs

Supporting Figure 11: Characterization and statistics of particle diameter at different grid-loading densities

Supporting Figure 12: Scheme for the transfection study mediated by eGFP-pDNA loaded scPNPs

Supporting Figure 13: Sustained effect of transfection mediated by eGFP-pDNA loaded scPNPs

Supporting Figure 14: Flow cytometry workflow and gating strategy for investigating population uptake of eGFP-pDNA loaded AF647-scPNPs over time.

Supporting Experimental Methods 1: Structure Illumination Microscopy

Supporting Experimental Methods 2: Nanoparticle Tracking Analysis (NTA)

Supporting Experimental Methods 3: Yield Calculations

Supporting Experimental Methods 4: Establishing a Correlation Factor between UV-vis Spectroscopy and NTA

Supporting Table S1: Uptake Efficiency Comparison with Established Nanocomplex Systems

Supporting Table S2: List of Abbreviations

Supporting References


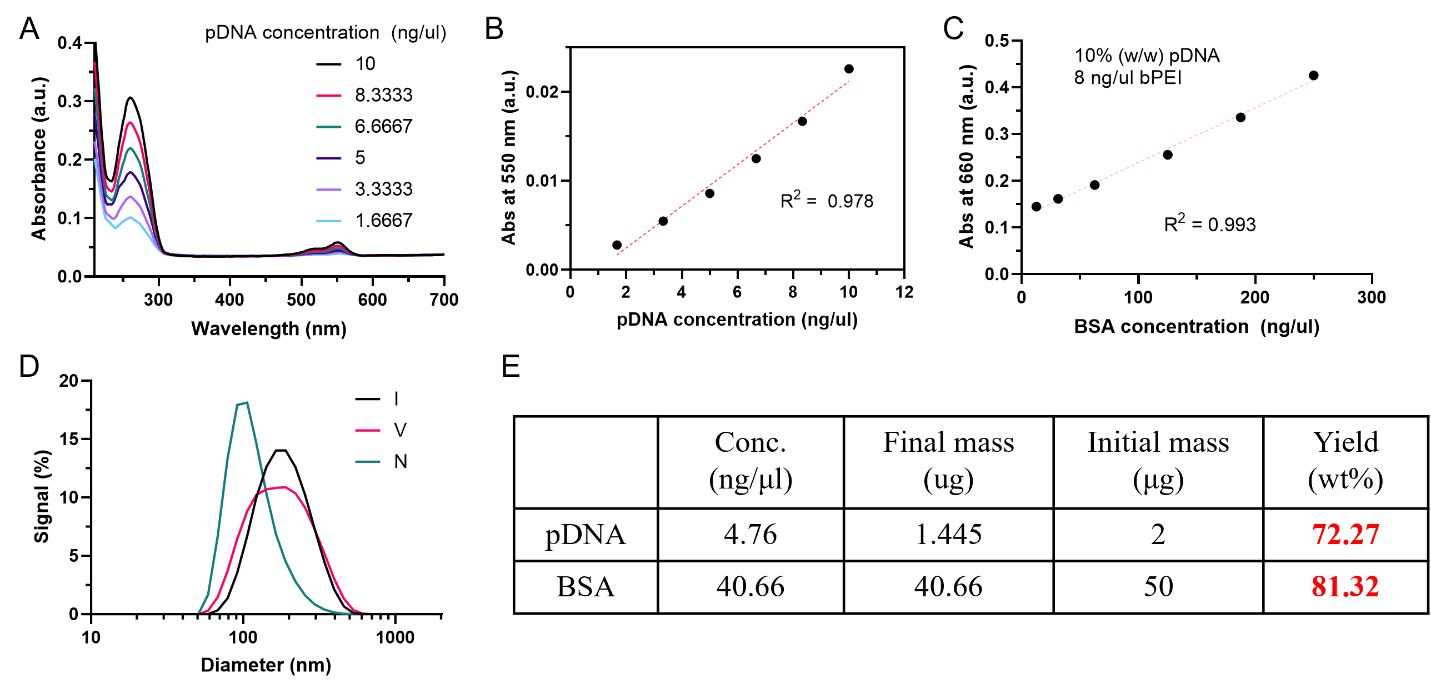


**Figure S1: Yield analysis on scPNPs loaded with Cyanine-3 (Cy3)-labeled eGFP-pDNA**. **(A)** UV-Vis absorbance was used to measure the Cy3-labeled eGFP-pDNA concentration. **(B)** Calibration curve for Cy3-labeled eGFP-pDNA. **(C)** A Pierce P660 assay was used to measure the protein concentration. **(D)** Diameter distributions for Cy3-labeled eGFP-pDNA scPNPs. **(E)** pDNA and protein mass before and after jetting and the resulting yield.


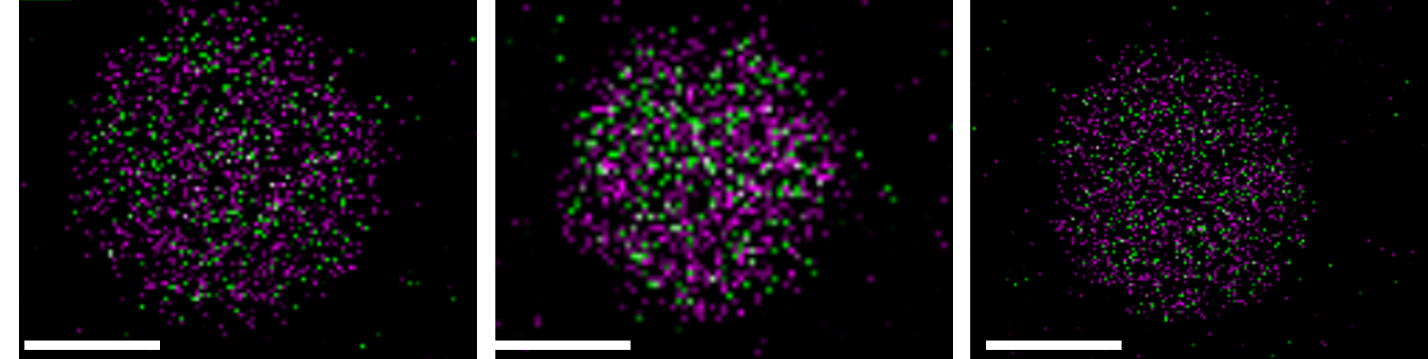


**Figure S2: Energy dispersive spectroscopy (EDS).** STEM-EDS of the spatial distribution of pDNA (10% w_pDNA_/w_albumin_) in as-jetted PNPs (Phosphorus = green, Nitrogen = pink). Scale bar 50 nm.


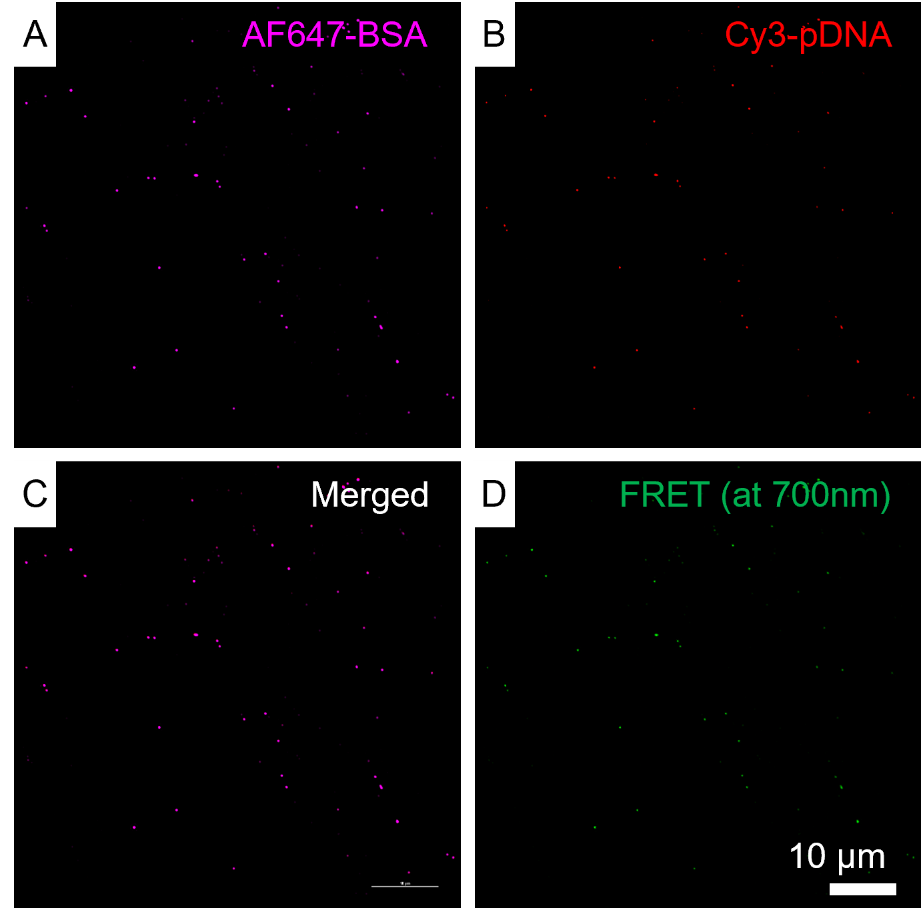


**Figure S3: Dual emission FRET study of AF647-labeled scPNPs loaded with Cy3-labeled eGFP-pDNA via structure illuminated microscopy (SIM, super-resolution).** **(A)** SIM image of scPNPs after PEI capping and redeposition, using AF647 emission. **(B)** SIM image of scPNPs showing Cy3 emission. **(C)** Merging of AF647 and Cy3 emission confirming co-localization of the pDNA and protein in the scPNPs. **(D)** FRET phenomenon between the Cy3 and AF647 in the scPNPs.


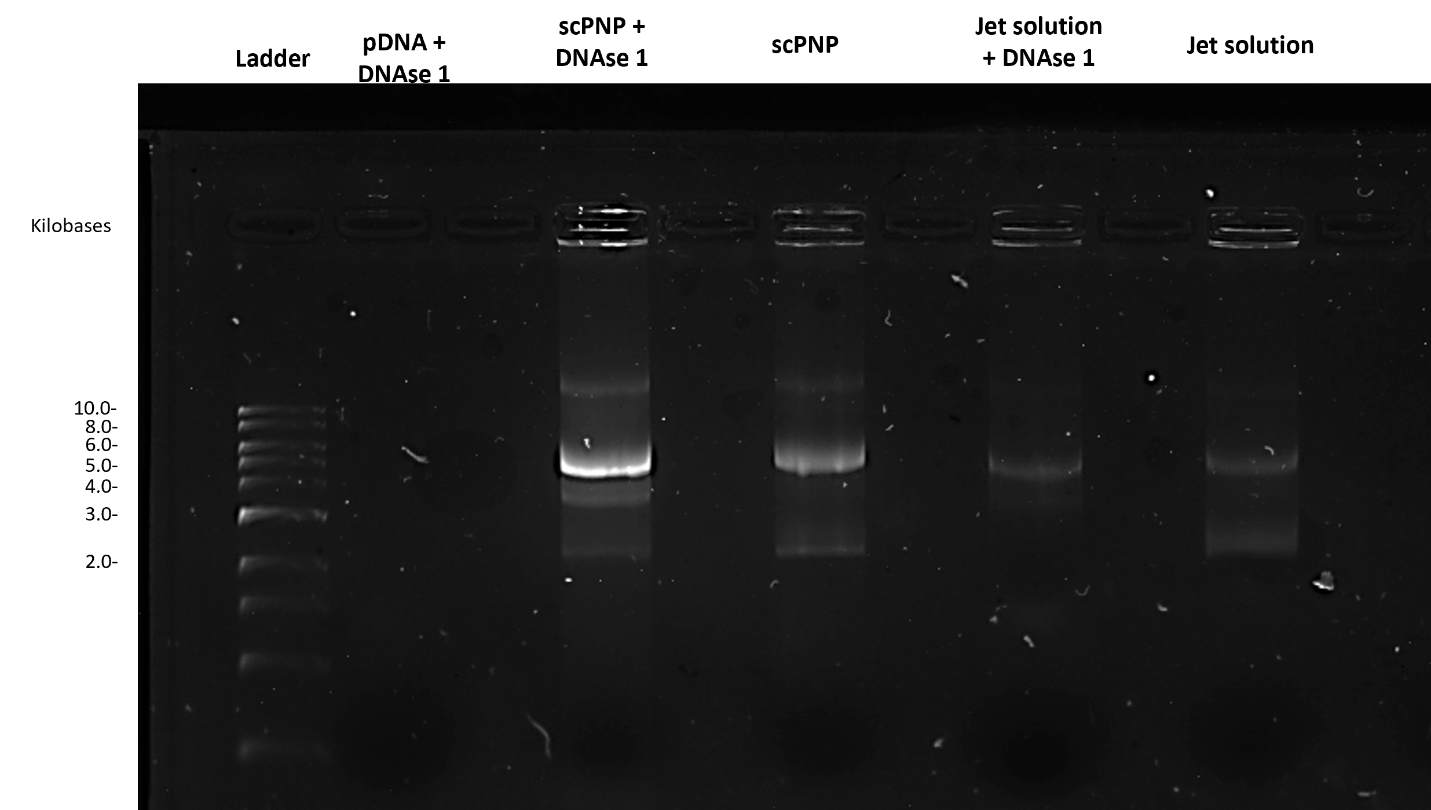


**Figure S4: Integrity of plasmid DNA in scPNPs versus a mixture of the same components.** Gel electrophoresis assay showing the pDNA bands in scPNPs and formulation mixture with and without incubation with DNAse1.


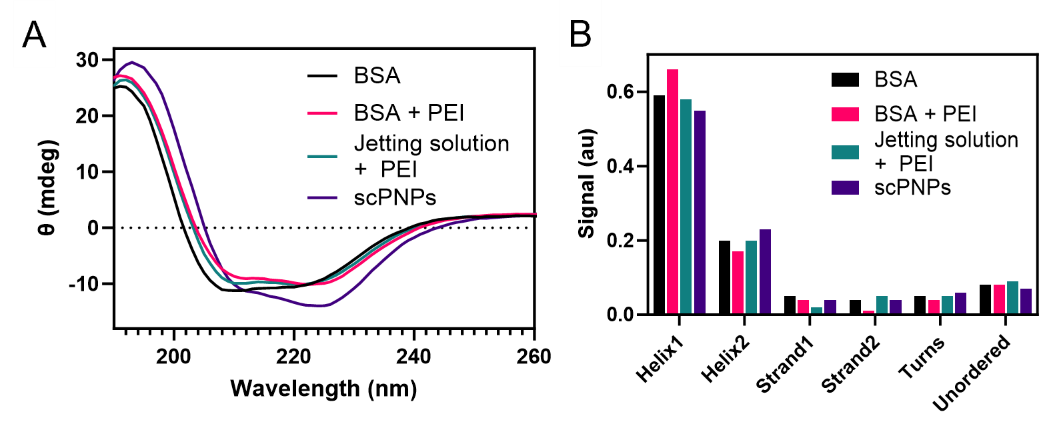


**Figure S5: CD spectroscopy of scPNPs and control groups.** **(A)** The impact on protein structure by jetting and PEI capping was assessed via circular dichroism (CD) spectroscopy and compared to control groups. **(B)** Analysis of the secondary structures performed by DichroWeb.

**
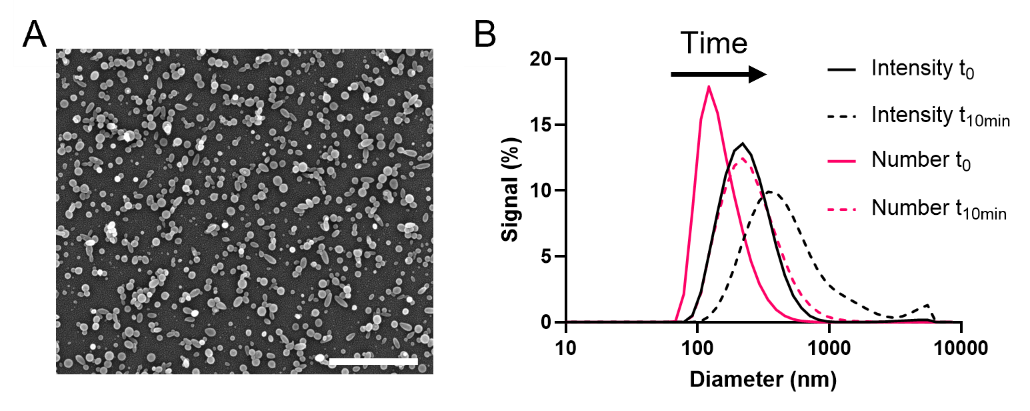
**

**Figure S6: Characterization of 70% (w_pDNA_/w_albumin_) eGFP-pDNA loaded scPNPs. (A)** SEM of as-jetted 70% eGFP-pDNA loaded scPNPs. Scale bar 1µm. **(B)** Diameter distribution of scPNPs immediately after PEI stabilization (solid lines) and after 10 minutes (dashed lines).


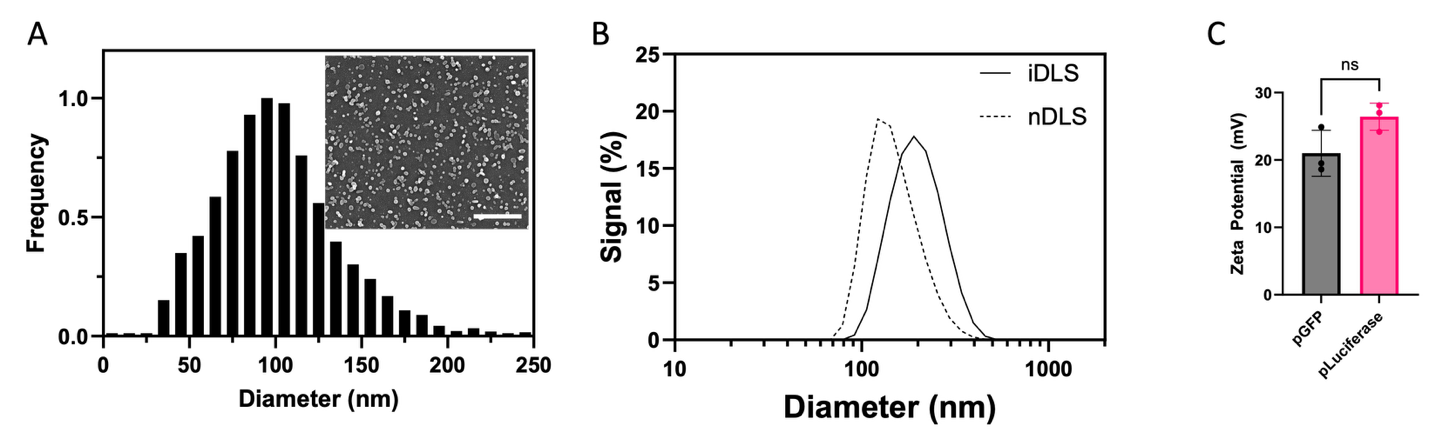


**Figure S7: Characterization of scPNPs encapsulating Luciferase-encoding plasmid.** **(A)** SEM and diameter distribution of as-jetted, dry-state protein nanoparticles, **(B)** Hydrodynamic diameter distribution of scPNPs. **(C)** Zeta potential of pLuciferase-scPNPs in comparison to pGFP-scPNPs.


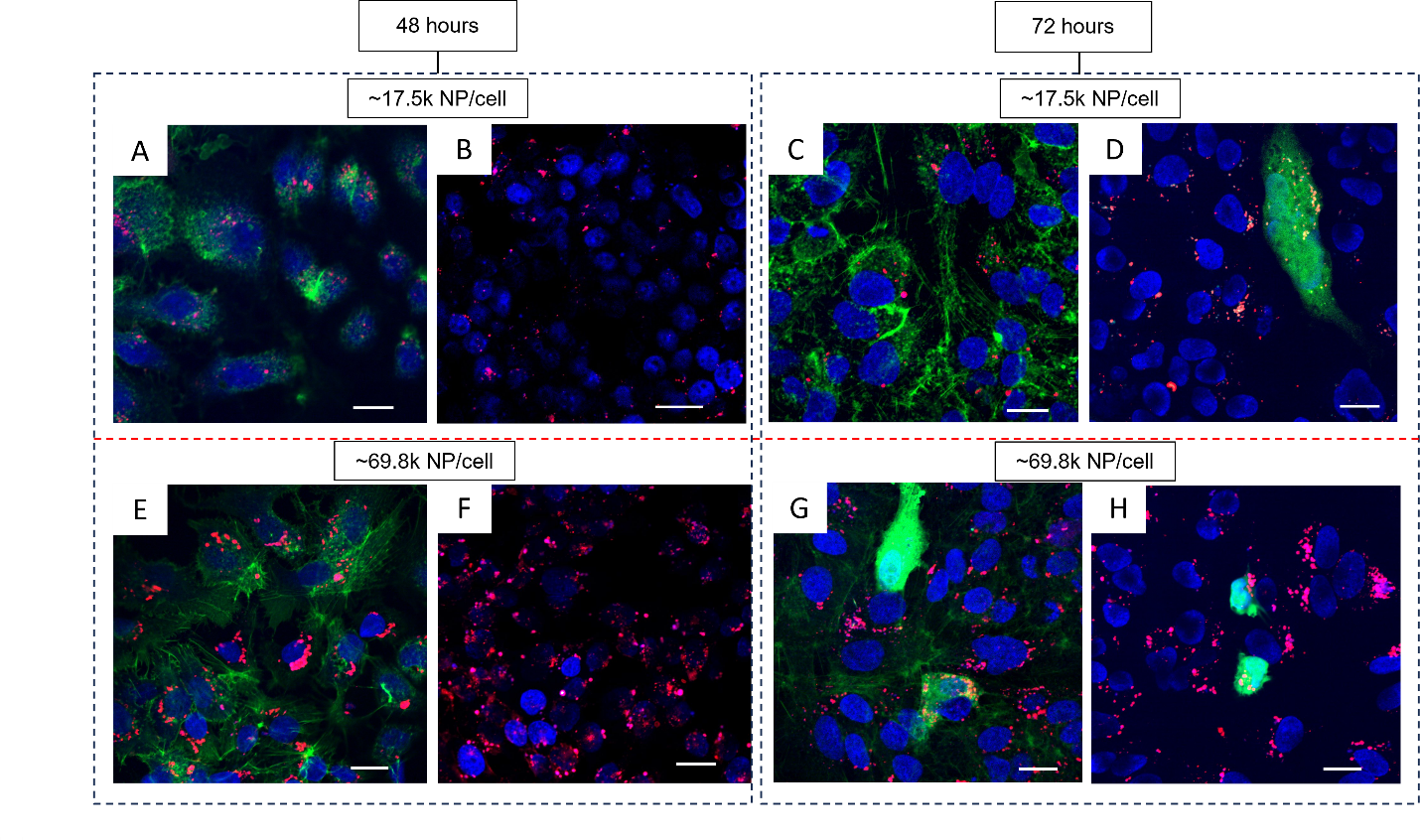


**Figure S8: Confocal images of HepG2 cells treated with high and medium dosages of scPNPs at 48 h and 72 h.** **(A-B)** HepG2 cells treated with medium dosage scPNPs (~17.5k) for 48 hours. **(C-D)** HepG2 cells treated with medium dosage scPNPs (~17.5k) for 72 hours. **(E-F)** HepG2 cells treated with medium dosage scPNPs (~69.8k) for 48 hours. **(G-H)** HepG2 cells treated with medium dosage scPNPs (~69.8k) for 72 hours. Blue = Nucleus, Red = scPNPs, Green = F-actin **(A, C, E, H)** and eGFP **(D, G, H)**.


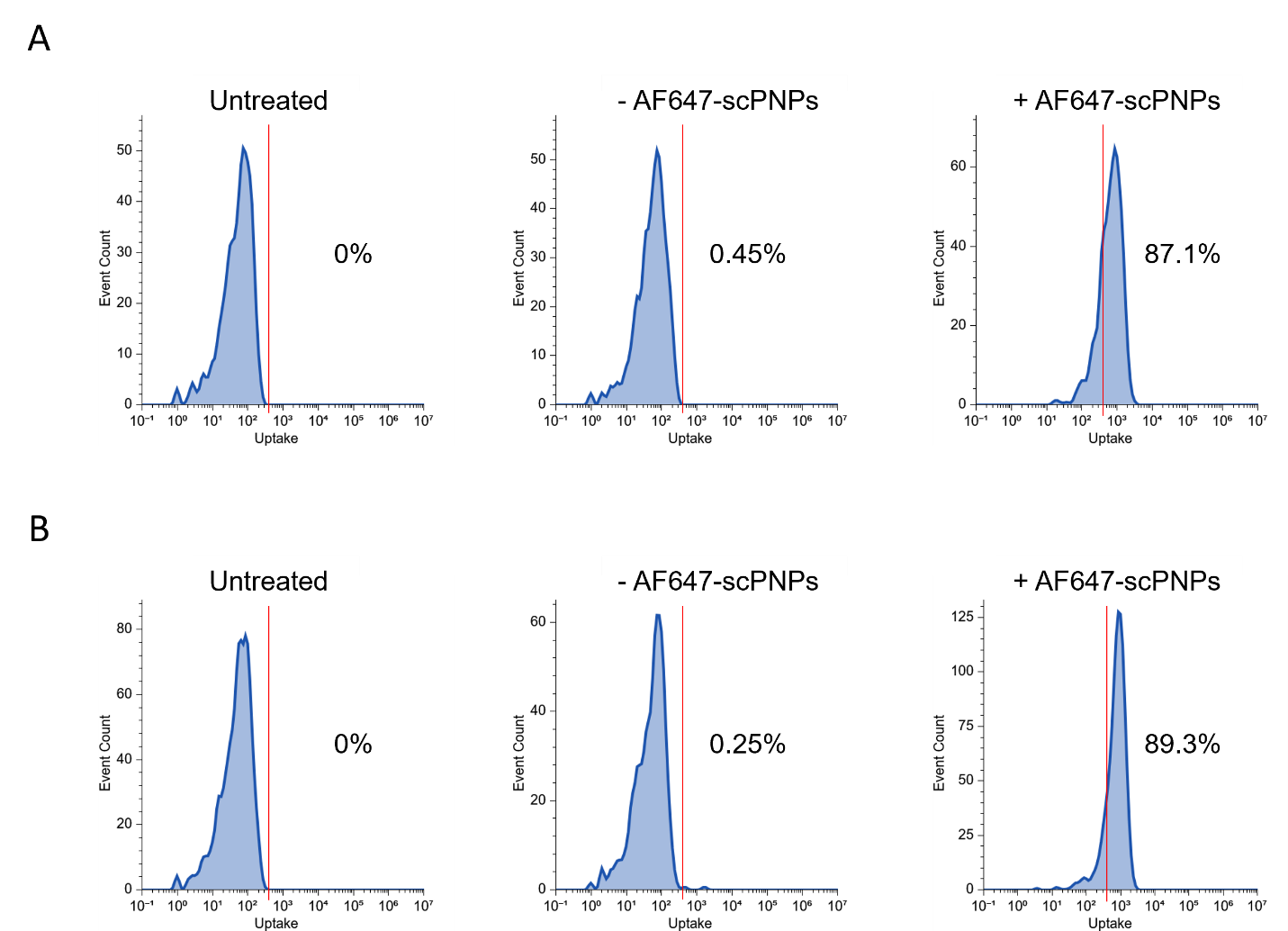


**Figure S9: Cellular uptake of AF647-labeled and unlabeled scPNPs after 1 hour of incubation. (A)** Flow cytometry of HepG2 cells treated with AF647 (-) and (+) scPNPs and **(B)** Flow cytometry of HEK293T cells treated with AF647 (-) and (+) scPNPs.


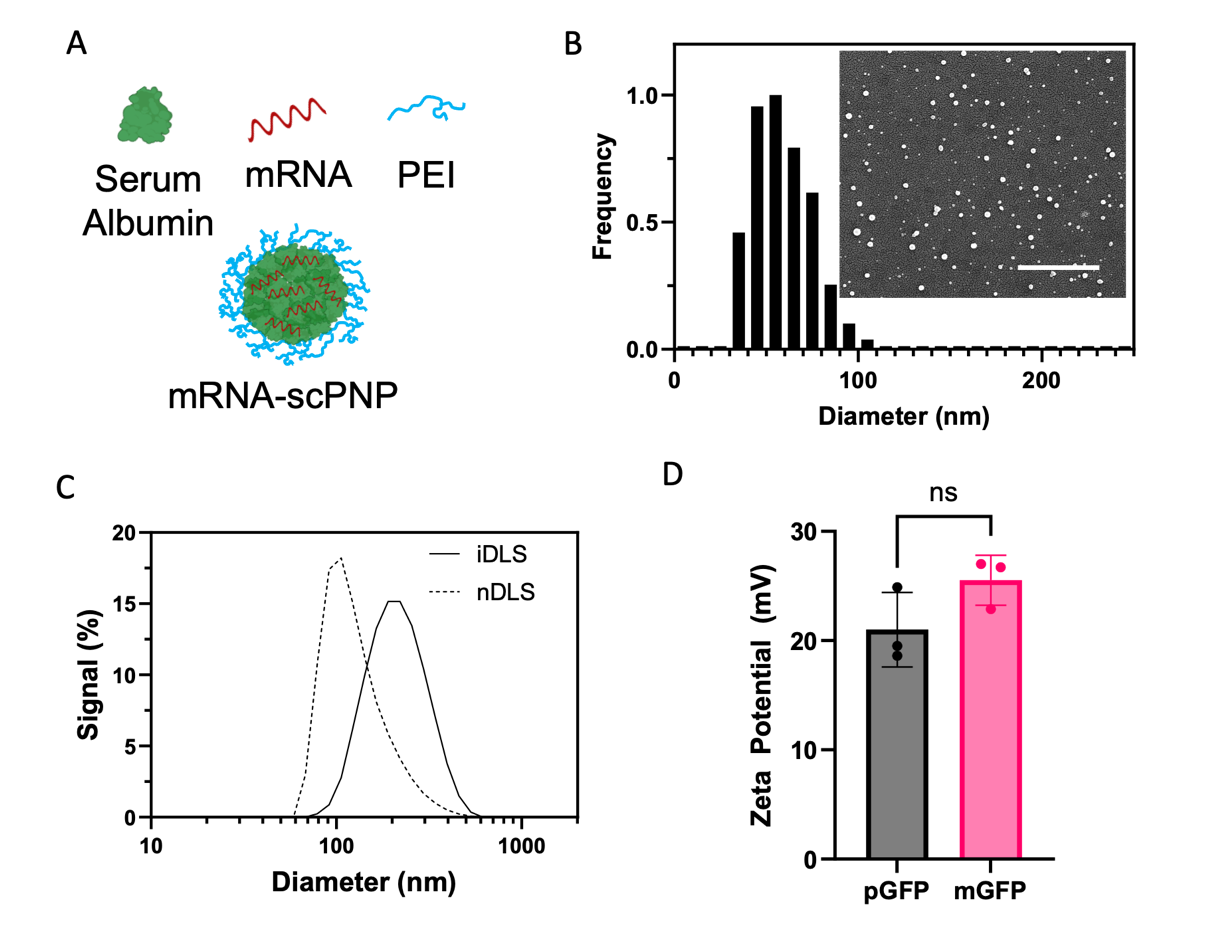


**Figure S10: Characterization of mRNA-loaded scPNPs.** **(A)** Illustration of the mRNA-scPNPs. **(B)** SEM and diameter distribution of the as-jetted, dry state protein nanoparticles. **(C)** Hydrodynamic diameter distribution of mRNA-scPNPs measured via DLS. **(D)** Zeta potential of mGFP-scPNPs in comparison to pGFP-scPNPs.


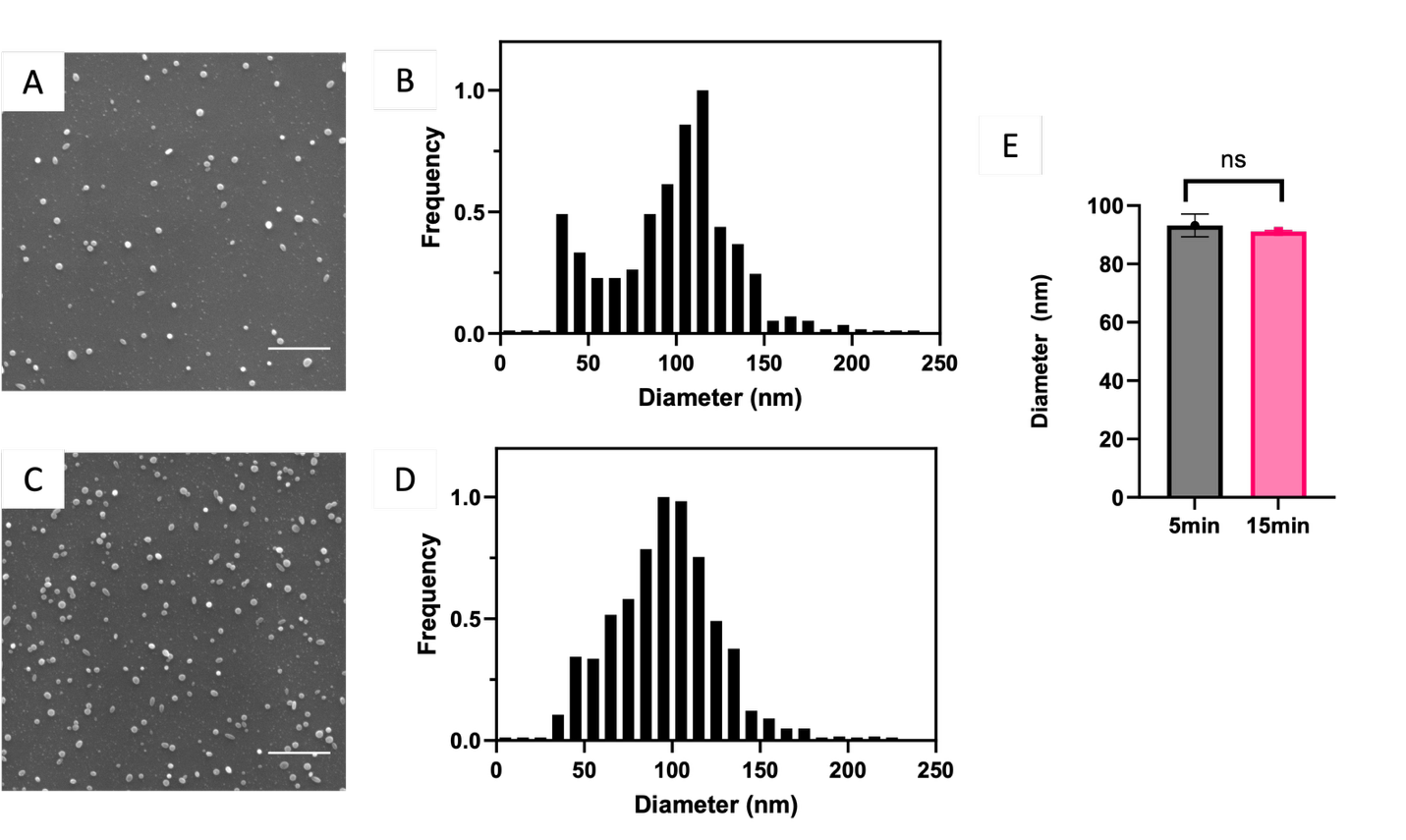


**Figure S11: Characterization and statistics of particle diameter at different grid-loading densities.** **(A)** Representative low-density SEM of 5 min of electrospray jetting, giving ~100 particles per micrograph. **(B)** Diameter distribution from 3 separate micrographs at 5 min jetting. **(C)** Representative low-density SEM of 15 min of electrospray jetting, giving ~200 particles per micrograph. **(D)** Diameter distribution from 3 separate micrographs at 15 min jetting. **(E)** Mean diameters are 93.2 ± 3.9 nm (5 min) and 91.1 ± 0.3 nm (15 min), with no statistically significant difference among the groups (t-test).


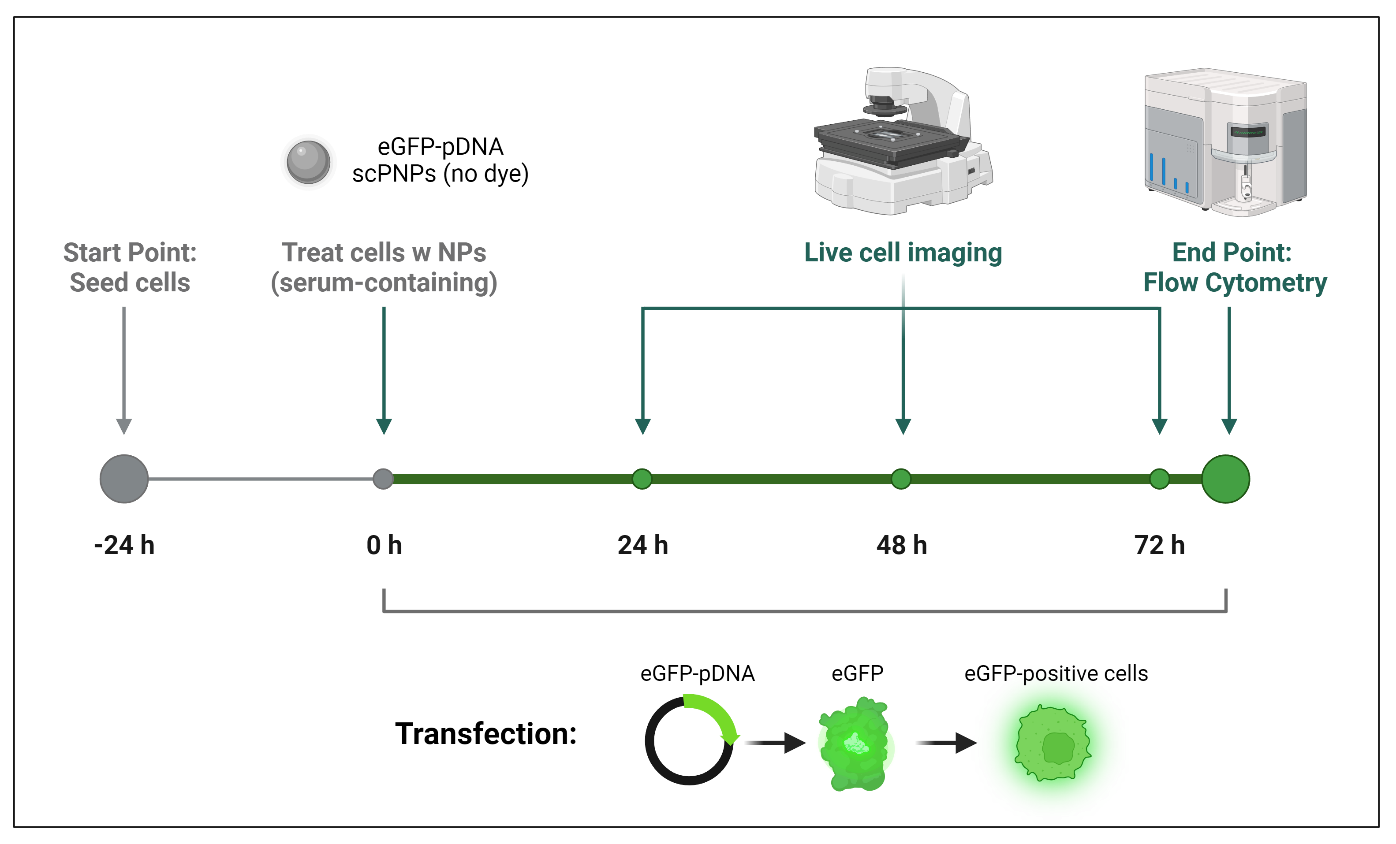


**Figure S12:** **Scheme for the transfection study mediated by eGFP-pDNA loaded scPNPs.** The eGFP-pDNA was utilized as the gene vector for better visualization of transfection activity in the transfection study. The EVOS M7000 Imaging System was utilized at 24, 48 and 72 h to observe the same cell culture plate, which can reveal the effects of scPNPs on the recipient cells in real-time; the Attune Flow Cytometer was utilized at the end point (72 h) after the live cell imaging to obtain high throughput data sets at the single-cell level. This established two-pronged workflow (imaging plus a high-throughput analysis) in our study ensures the highest confidence in the results. Figure S12 created with [BioRender.com](https://biorender.com/).


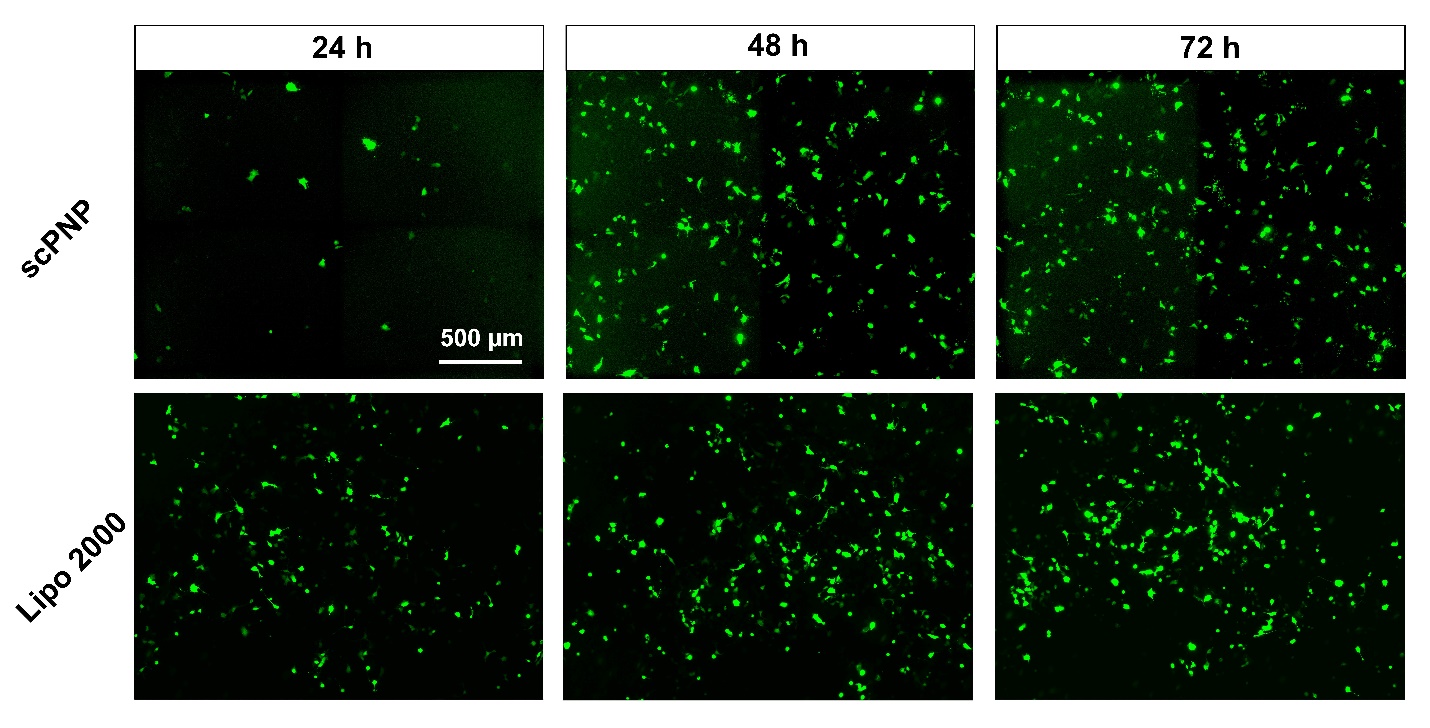


**Figure S13: Sustained effect of transfection mediated by eGFP-pDNA loaded scPNPs.** Representative live cell images of HepG2 cells, 24, 48, and 72 h post-transfection mediated by eGFP-pDNA-loaded scPNPs (upper row) and Lipofectamine 2000 (bottom row). Scale bar indicates 500 µm.


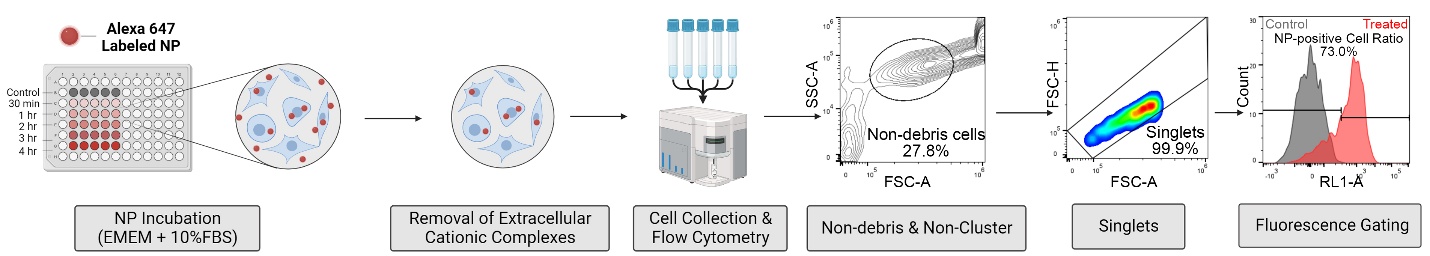


**Figure S14:** **Flow cytometry workflow and gating strategy for investigating population uptake of AF647-scPNPs over time.** All scPNPs used under this experimental setup for uptake studies were loaded with 10wt% eGFP-pDNA as a model non-viral gene vector. Figure S14 created with [BioRender.com](https://biorender.com/).

**Supporting Experimental Methods 1:** **Structure Illumination Microscopy**

Co-localization of the fluorescent AF647-scPNPs and Cy3-pDNA were confirmed using structure illumination microscopy (SIM). scPNP samples were drop-casted and mounted onto microscope slides. Imaging was performed with the University of Michigan Microscopy and Image Analysis Laboratory (MIL) Core’s Nikon N-SIM +A1R microscope and a 100x objective with collection occurring at Hamamatsu Orca-Flash 4.0 detector.

**Supporting Experimental Methods 2: Nanoparticle Tracking Analysis (NTA)**

The scPNP concentration was acquired using a Malvern Nanosight NS300 equipped with a 488-laster light source. Samples were diluted accordingly in H_2_O. Measurements were recorded at 25^o^C using continuous flow mode. Five captures of 60 s each were used to produce the movie files that were then assessed by NTA to generate concentration data to determine the number of NPs per volume.

**Supporting Experimental Methods 3: Yield Calculations**

To calculate the yield of the scPNP fabrication process, both the yield of the Cy3-pDNA as well as the yield of the protein were measured, using UV-vis absorbance and a Pierce 660 protein assay, respectively. First, a calibration curve with known concentration of Cy3-pDNA was created to obtain a concentration value for a known absorbance value. Additionally, a calibration curve was created for the Pierce 660 protein assay which included known concentrations of both PEI and DNA in addition to known concentrations of BSA. After both calibration curves were established, the Cy3-pDNA loaded scPNPs were quantified for mass yield of pDNA and of BSA.

**Supporting Experimental Methods 4: Establishing a Correlation Factor between UV-vis Spectroscopy and NTA**

To establish a relationship between DNA mass and scPNP mass, we measured the absorbance of the Cy3-pDNA scPNPs and the NP number per volume at the same dilution factors. First, a calibration curve with known concentration of Cy3-pDNA was created to obtain a concentration value for a known absorbance value for a given dilution of scPNP samples. Then, we established a workflow to calculate the scPNP mass concentration from the measured NP number per volume from NTA (Equations 1-5). To do this, we estimated a density for the scPNP based on the densities of each component and their respective weight fractions (Eq 1).

$\rho_{scPNP}= {(\rho}_{albumin}*\frac{{\%}_{albumin}\frac{w}{w}}{{\%}_{albumin}+{\%}_{pDNA}+{\%}_{PEI}})+(\rho_{pDNA}*\frac{{\%}_{pDNA}\frac{w}{w}}{{\%}_{albumin}+{\%}_{pDNA}+{\%}_{PEI}})+{(\rho}_{PEI}*\frac{{\%}_{PEI}\frac{w}{w}}{{\%}_{albumin}+{\%}_{pDNA}+{\%}_{PEI}})$ (Eq 1)

Then, we used the scPNP diameter to calculate the scPNP volume for a given distribution (Eq 2). Using the calculated density (Eq 1, we then calculated the scPNP mass (Eq 3). The average scPNP mass was calculated using Equation 4.

$Particle volume=\frac{4}{3}\pi({\frac{d_{scPNP}}{2})}^{3}$ (Eq 2)

$Particle mass=Particle volume*\rho_{scPNP}$ (Eq 3)

$Particle mass =\sum(Particle mass*\frac{Particle number}{bin})$ (Eq 4)

Knowing the Cy3-pDNA concentration and the scPNP concentration for each dilution, we established a correlation factor between the two (Equation 5). The correlation factor is an average result of three batch repeats. This correlation factor was used to determine the pDNA concentration from the measured scPNP concentration with NTA.

$\frac{{Cy3}_{pDNA} (\frac{ng}{\mu l})}{scPNP concentration (\frac{ng}{\mu l})}=1.11*{10}^{-5}\pm0.07*{10}^{-5}$ (Eq 5)

**Supporting Experimental Methods 5: Determination of Average Plasmids in scPNPs**

The average number of plasmid molecules per scPNP were calculated using Equations 6-9. First, the particle diameter (d) was obtained from nDLS and it was used to calculate the particle volume using Equation 2 above. Next, the total mass in each bin was calculated using the volume and density (calculated in Eq 1 above) and the nDLS counts for each bin. DNA mass per bin was calculated using the DNA loading fraction and total mass of particle (Eq 7). The number of molecules per particle was calculated using Equation 8. And lastly, the average number of plasmid molecules per scPNP was obtained using Equation 9.

$\frac{Total mass}{bin}=\left( Particle volume*\rho_{scPNP} \right)* N_{DLS}$ (Eq 6)

$\frac{DNA mass}{bin}=\frac{\frac{Total mass}{bin}}{DNA fraction}$ (Eq 7)

$\frac{DNA molecule}{particle}=\frac{(\frac{\frac{DNA mass}{bin}}{{DNA}_{Molecular Weight}})*6.02E23}{N_{DLS}}$ (Eq 8)

$\frac{Average plasmid}{scPNP}=\frac{\sum(N_{DLS}*\frac{DNA molecule}{particle})}{\sum N_{DLS}}$ (Eq 9)

**Supporting Table S1: Uptake Efficiency Comparison with Established Nanoparticle Systems**

| **Nanocomplex System** | **Cell Type** | **DNA dosage (ng)*** | **Cell number**** | **w_carrier_/w_DNA_** | **Dosage of NPs (ng/cell)** | **Uptake Efficiency (%)***** | **Time point**  **(hours)** | **References** |
| --- | --- | --- | --- | --- | --- | --- | --- | --- |
| Fluo-pDNA  /T704/peptide | 16HBE | 133 | 2.5x10^4^ | 63:1 | 0.34 | 58.80 | 4 | Nature Nano., *2019^[1]^* |
| PBAE447/pDNA | glioblastoma | 473 | 1.6x10^4^ | 30:1 | 0.93 | 71.25 | 4 | Acta Biomater., *2016^[2]^* |
| PBAE536/pDNA | 1 hepatocyte & 8 HCC cell lines**** | 600 | 1.0 x10^4^ | 25:1 | 1.56 | 99.00 | 2 | J. Control Release, *2017^[3]^* |
| PBAE457/pDNA | COS-7 | 600 | 1.5 x10^4^ | 60:1 | 2.44 | 95.92 | 4 | Mol. Pharmaceutics, *2012^[4]^* |
| PEI /pDNA | 293T | 116 | 4.4 x10^4^ | 0.4:1 | 0.0037 | 90.00 | 4 | J. Control Release, *2011^[5]^* |
| Hydroquinine/pDNA | 293T | 168 | 8.4 x10^3^ | 9:1 | 0.2 | 91.00 | 24 | JACS Au, *2023^[6]^* |
| Cell membrane/PEI/pDNA | C6 | 27.43 | 1.8 x10^3^ | 21:1 | 0.033 | 72.00 | 24 | J. Control Release, *2021^[7]^* |
| scPNP/pDNA | HepG2 | 261 | 2.0 x10^4^ | 10:1 | 0.1436 | 98.00 | 4 | **Our Work** |
|  | 293T |  |  |  |  | 94.48 | 4 |  |

*ng per well, after normalization to the 96-well cell culture plate;

**cells per well, after normalization to the 96-well cell culture plate;

*** Uptake efficiency, defined as the ratio of NP-positive cells to the total cells, was evaluated *via* flow cytometry 4h post incubation;

**** A human hepatocyte line (THLE-3 [ATCC® CRL-11233™]) and eight HCC cell lines (Hep3b [ATCC® HB-8064™], HepG2 [ATCC® HB-8065™], C3A, SK-HEP-1, PLC/PRF/5, SNU-387, SNU-423, SNU-475 were tested in this study.

**Supporting Table S2: List of Abbreviations**

| **Abbreviation** | **Full Name** |
| --- | --- |
| NP | nanoparticle |
| PNP | protein-based nanoparticle |
| scPNP | surface-capped protein nanoparticle |
| pDNA | plasmid DNA |
| mRNA | messenger RNA |
| PEI | polyethylenimine |
| bPEI | branched PEI |
| LNP | lipid nanoparticle |
| EHD | electrohydrodynamic |
| MW | molecular weight |
| SEM | scanning electron microscopy |
| DLS | dynamic light scattering |
| nDLS | number DLS |
| iDLS | intensity DLS |
| PDI | polydispersity index |
| Cy-3 | cyanine-3 |
| eGFP | enhanced green fluorescent protein |
| NTA | nanoparticle tracking analysis |
| TEM | transmission electron microscopy |
| EDS | energy dispersive spectroscopy |
| SIM | structure illumination microscopy |
| AF647 | Alexa Fluor-647 |
| BSA  FRET | bovine serum albumin  fluorescence resonance energy transfer |
| SD  CD | standard deviation  circular dichroism |
| ns | no significance |
| CME | clathrin-mediated endocytosis |
| EIPA | 5-(N-ethyl-N-isopropyl) amiloride |
| MβCD | methyl-β-cyclodextrin |

**Supporting References**

[1] S. Guan, A. Munder, S. Hedtfeld, P. Braubach, S. Glage, L. Zhang, S. Lienenklaus, A. Schultze, G. Hasenpusch, W. Garrels, *Nature nanotechnology* **2019**, 14, 287.

[2] C. J. Bishop, R. L. Majewski, T.-R. M. Guiriba, D. R. Wilson, N. S. Bhise, A. Quiñones-Hinojosa, J. J. Green, *Acta biomaterialia* **2016**, 37, 120.

[3] C. G. Zamboni, K. L. Kozielski, H. J. Vaughan, M. M. Nakata, J. Kim, L. J. Higgins, M. G. Pomper, J. J. Green, *Journal of Controlled Release* **2017**, 263, 18.

[4] J. C. Sunshine, D. Y. Peng, J. J. Green, *Molecular pharmaceutics* **2012**, 9, 3375.

[5] Y. Yue, F. Jin, R. Deng, J. Cai, Y. Chen, M. C. Lin, H.-F. Kung, C. Wu, *Journal of controlled release* **2011**, 155, 67.

[6] P. Roy, N. W. Kreofsky, M. E. Brown, C. Van Bruggen, T. M. Reineke, *JACS Au* **2023**, 3, 1876.

[7] a)P. Roy, N. W. Kreofsky, T. M. Reineke, *Biomacromolecules* **2024**, 25, 6693; b)Y. Yue, F. Jin, R. Deng, J. Cai, Y. Chen, M. C. Lin, H.-F. Kung, C. Wu, *Journal of controlled release* **2011**, 155, 67.
